# Supplementary material for: C-C Motif Chemokine Ligand 5 (CCL5) Promotes Irradiation-Evoked Osteoclastogenesis
Source: Int J Mol Sci. 2023 Nov 10;24(22):16168. doi: 10.3390/ijms242216168 (PMC10671276; doi:10.3390/ijms242216168)

C-C Motif Chemokine Ligand 5 (CCL5) Promotes Irradiation-Evoked Osteoclastogenesis

Jing Wang, Fanyu Zhao, Linshan Xu, Jianping Wang, Jianglong Zhai, Li Ren And Guoying Zhu*

Department of Radiological Hygiene, Institute of Radiation Medicine, Fudan University, 2094 Xietu Road, Shanghai 200032, China;
* Correspondence: zhugy@shmu.edu.cn; Tel.: +86-21-64049847

Supplementary Materials

- **The process for obtaining tiny single cut-out lanes from the complete gels**

Figure1:

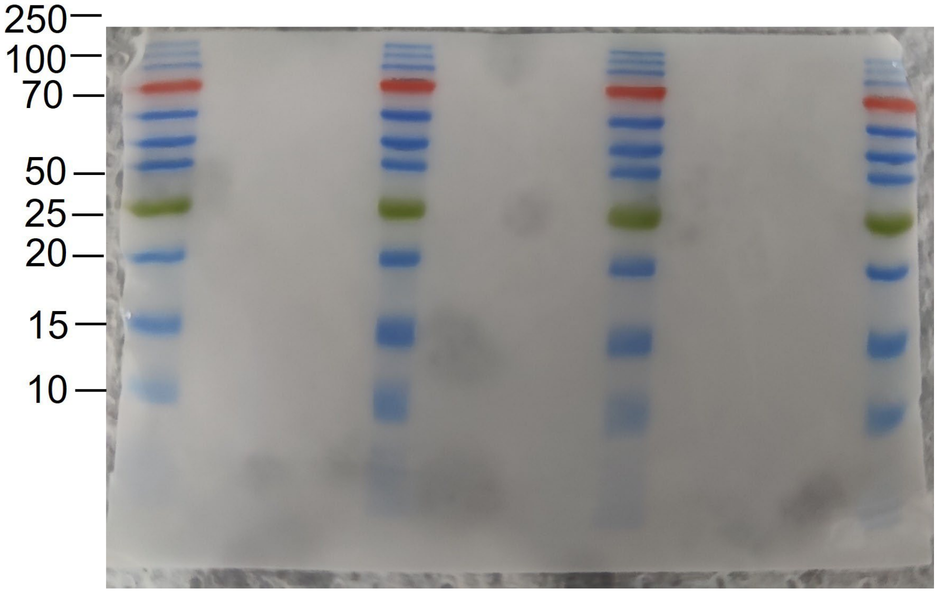

As shown in Figure 1, the molecular weight range of this complete blot is 10~250kDa, and concentration of the gel was 15%. It was used for the detection of CCL5 (10kDa) and β-actin (42kDa). After transfer and blocking, we cut the complete blot into strips in strict accordance with the pre-stained marker position for subsequent antibody incubation and detection of the target protein band separately.

Figure2:

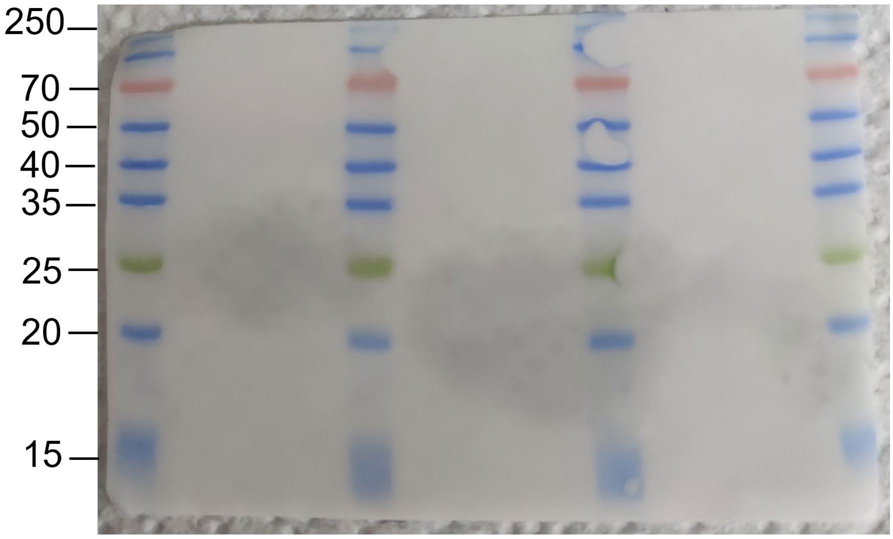

As shown in Figure 2, the molecular weight range of this complete blot is 15~250kDa, and concentration of the gel was 12.5%. It was used for the detection of p21 (21kDa), p16 (16kDa) and β-actin (42kDa). After transfer and blocking, we cut the complete blot into strips in strict accordance with the pre-stained marker position for subsequent antibody incubation and detection of the target protein band separately.

Figure3:

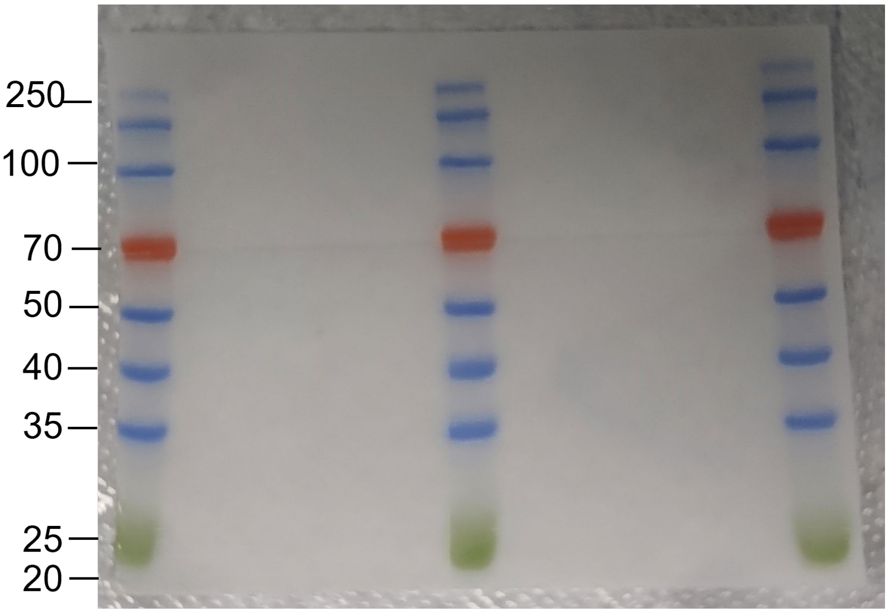

As shown in Figure 3, the molecular weight range of this complete blot is 20~250kDa, and concentration of the gel was 10%. It was used for the detection of E11 (36kDa), RANKL (35kDa), OPG (46kDa), DMP1 (34kDa) γ-H2AX (21kDa) and β-actin (42kDa). After transfer and blocking, we cut the complete blot into strips in strict accordance with the pre-stained marker position for subsequent antibody incubation and detection of the target protein band separately.

Figure4:

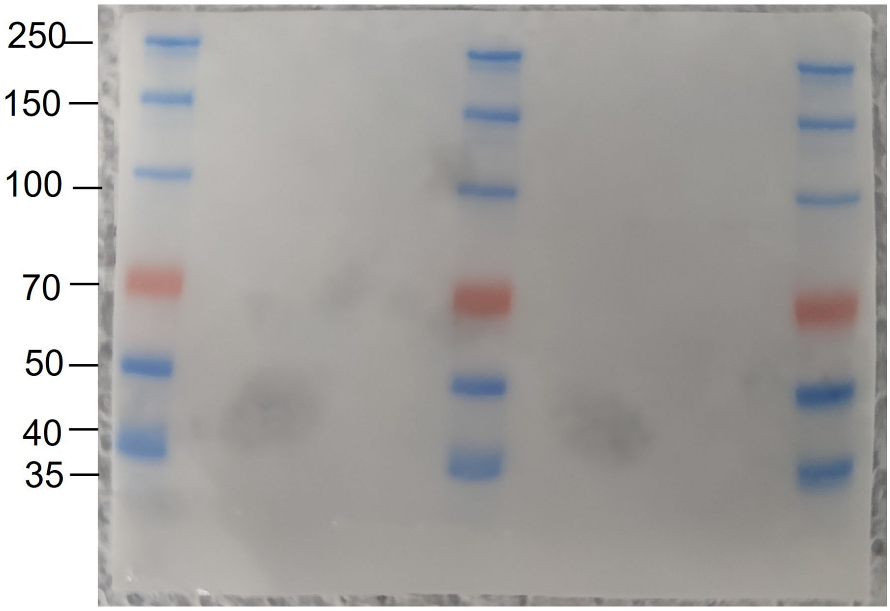

As shown in Figure 4, the molecular weight range of this complete blot is 35~250kDa, and concentration of the gel was 7.5%. It was used for the detection of p-JAK1 (130kDa), t-STAT3 (88kDa), p-STAT3 (88kDa) protein, and the internal reference protein β-actin (42kDa). After transfer and blocking, we cut the complete blot into strips in strict accordance with the pre-stained marker position for subsequent antibody incubation and detection of the target protein band separately.

Supplementary note:

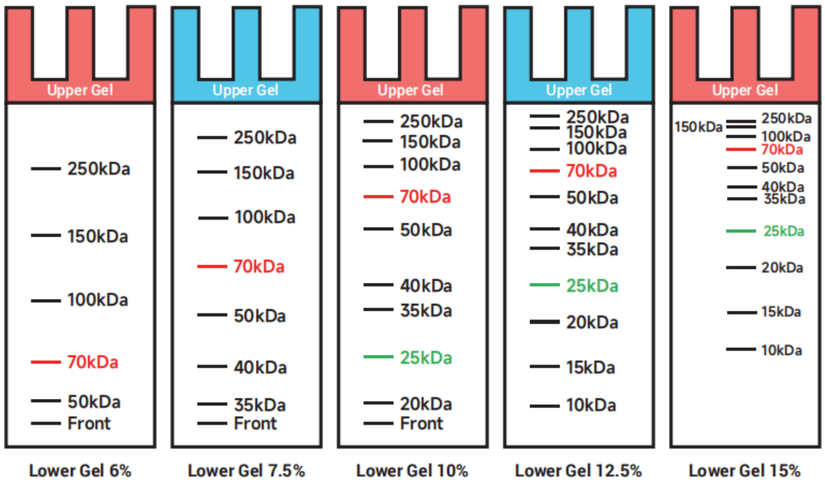

The picture below is the complete picture after transfer and standard images provided by manufacturer. The whole blot was blocked with 5% BSA for 30 minutes at room temperature, and then we cut it into strips according to the pre-stained marker position. In the future we will pay attention to showing molecular weight markers with immunoblot panels.

● Original blots of western blotting assays

Figure 1B. Original blots of β-actin proteins.

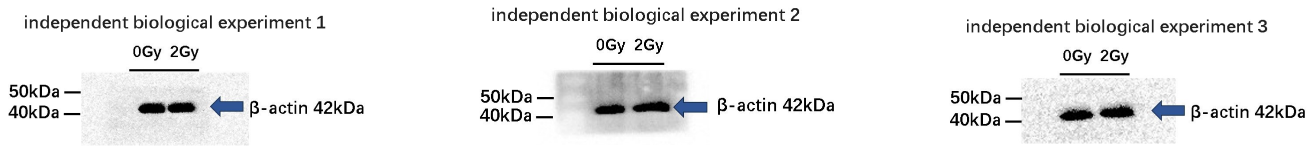

Figure 1B. Original blots of E11 proteins.

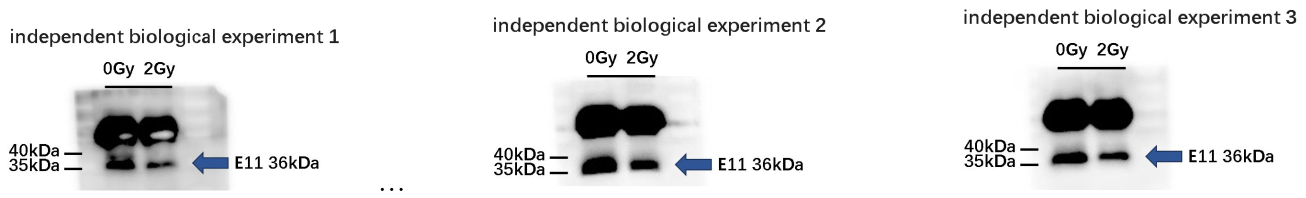

Figure 1C. Original blots of β-actin proteins.

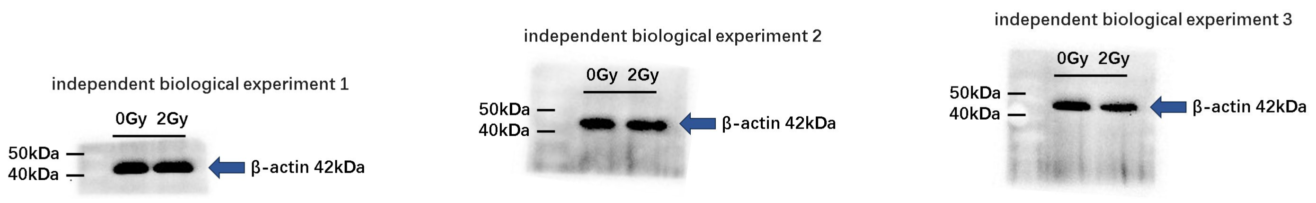

Figure 1C. Original blots of RANKL proteins.

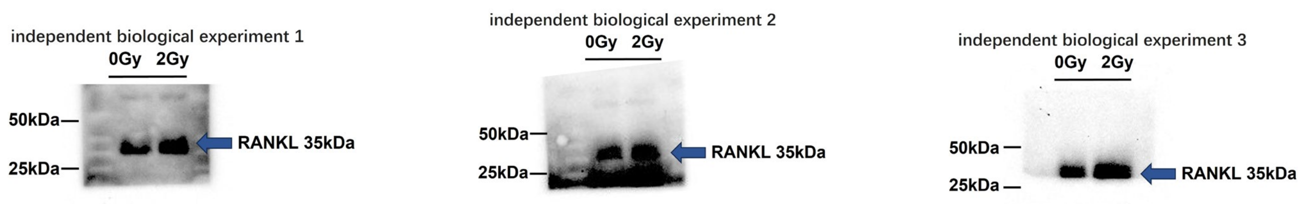

Figure 1C. Original blots of OPG proteins.

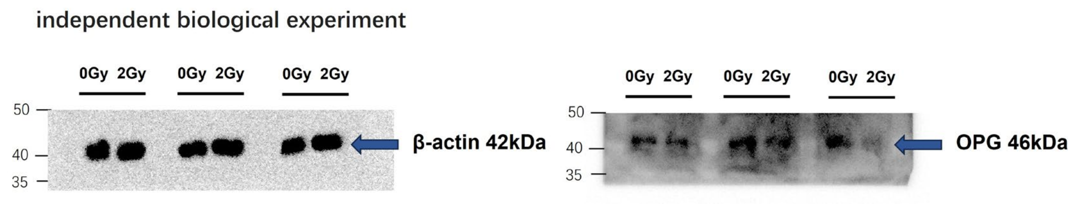

Figure 1C. Original blots of DMP1 proteins.

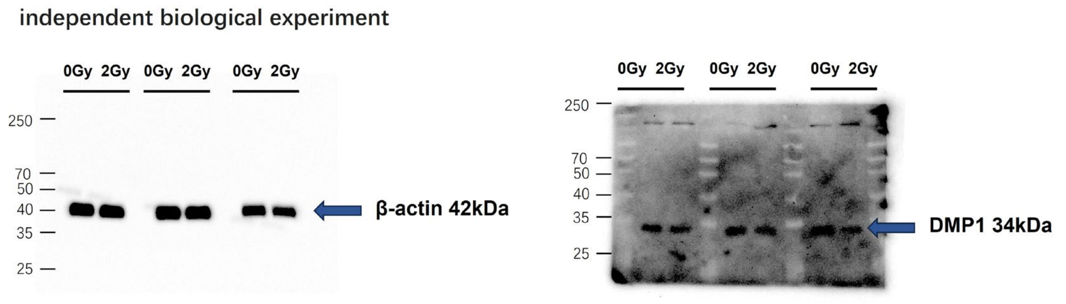

Figure 2B. Original blots of β-actin proteins.

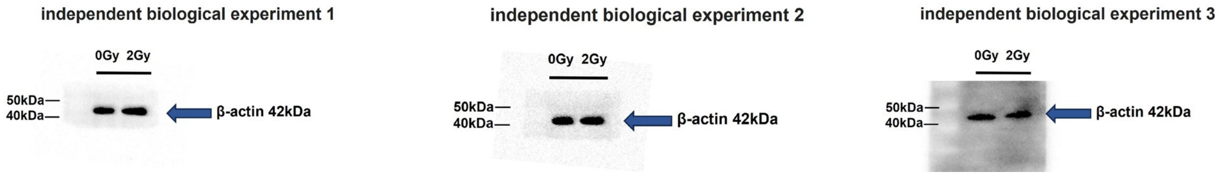

Figure 2B. Original blots of γ-H2AX proteins.

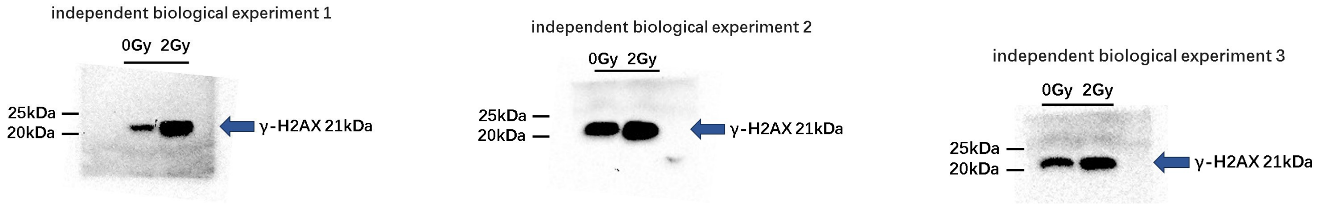

Figure 2C. Original blots of β-actin proteins.

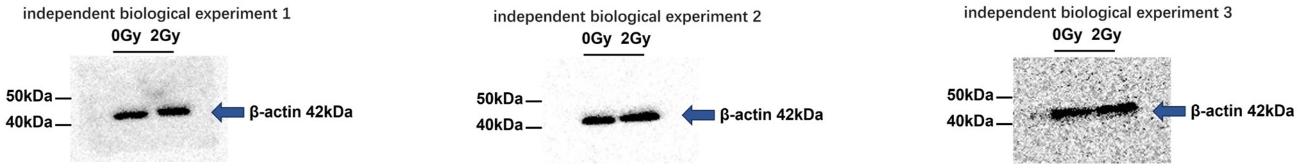

Figure 2C. Original blots of p16 proteins.

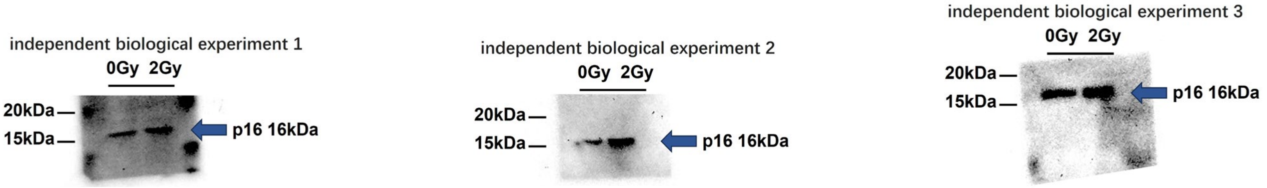

Figure 2C. Original blots of β-actin proteins.

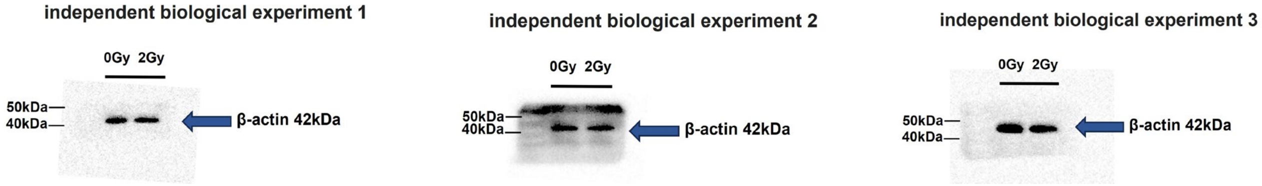

Figure 2C. Original blots of p21 proteins.

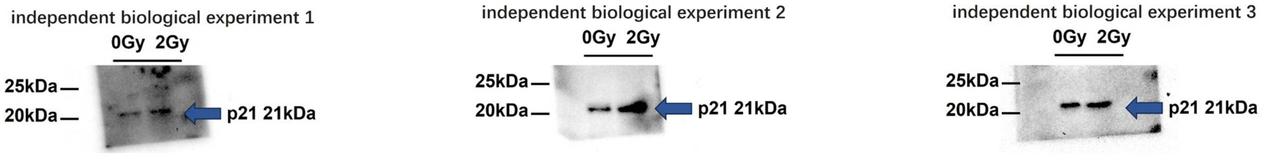

Figure 3C. Original blots of CCL5 proteins.

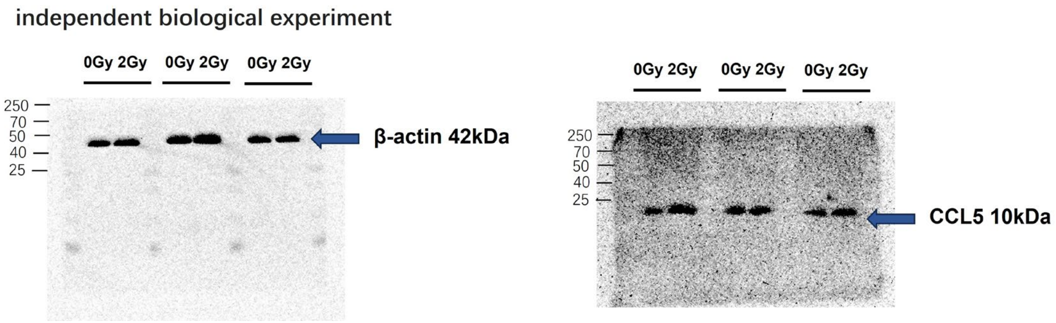

Figure 6A. Original blots of p-STAT3 proteins.

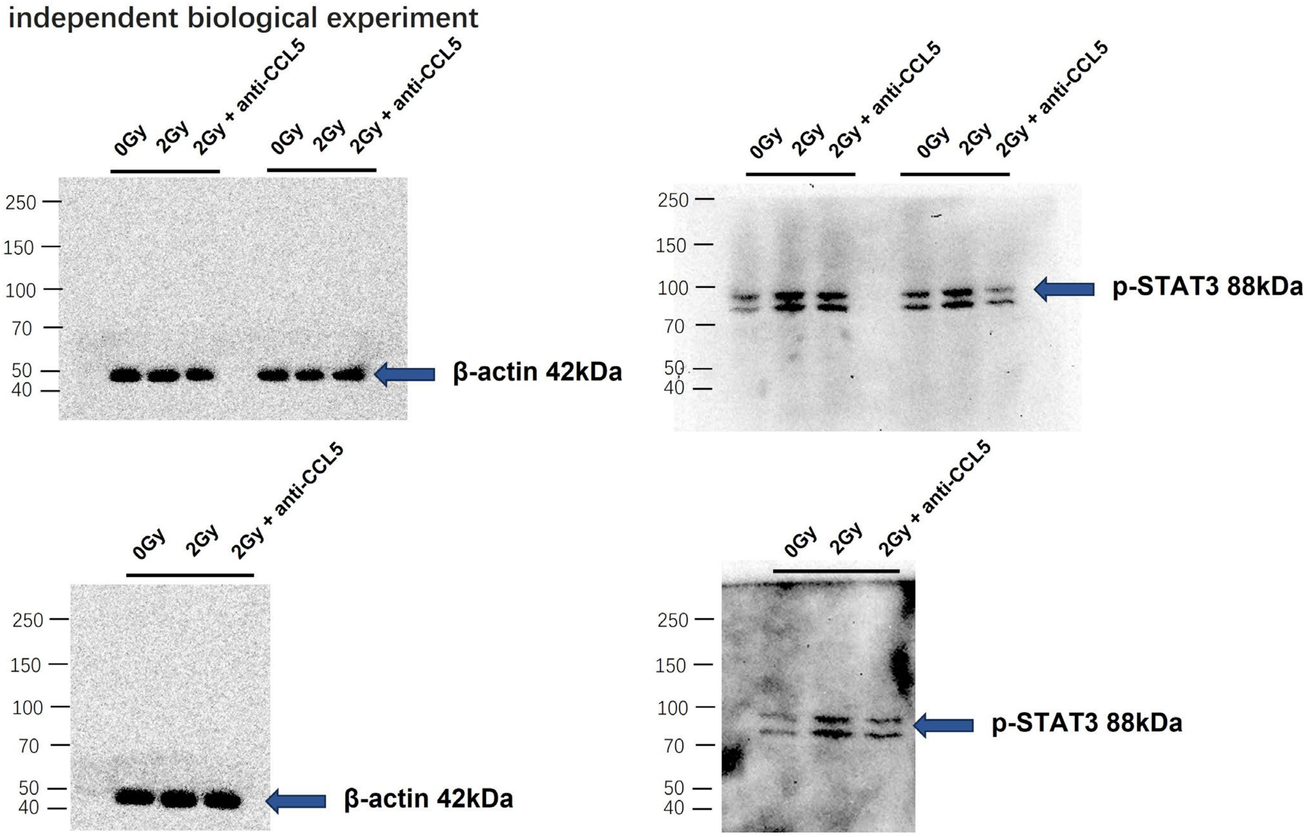

Figure 6A. Original blots of t-STAT3 proteins.

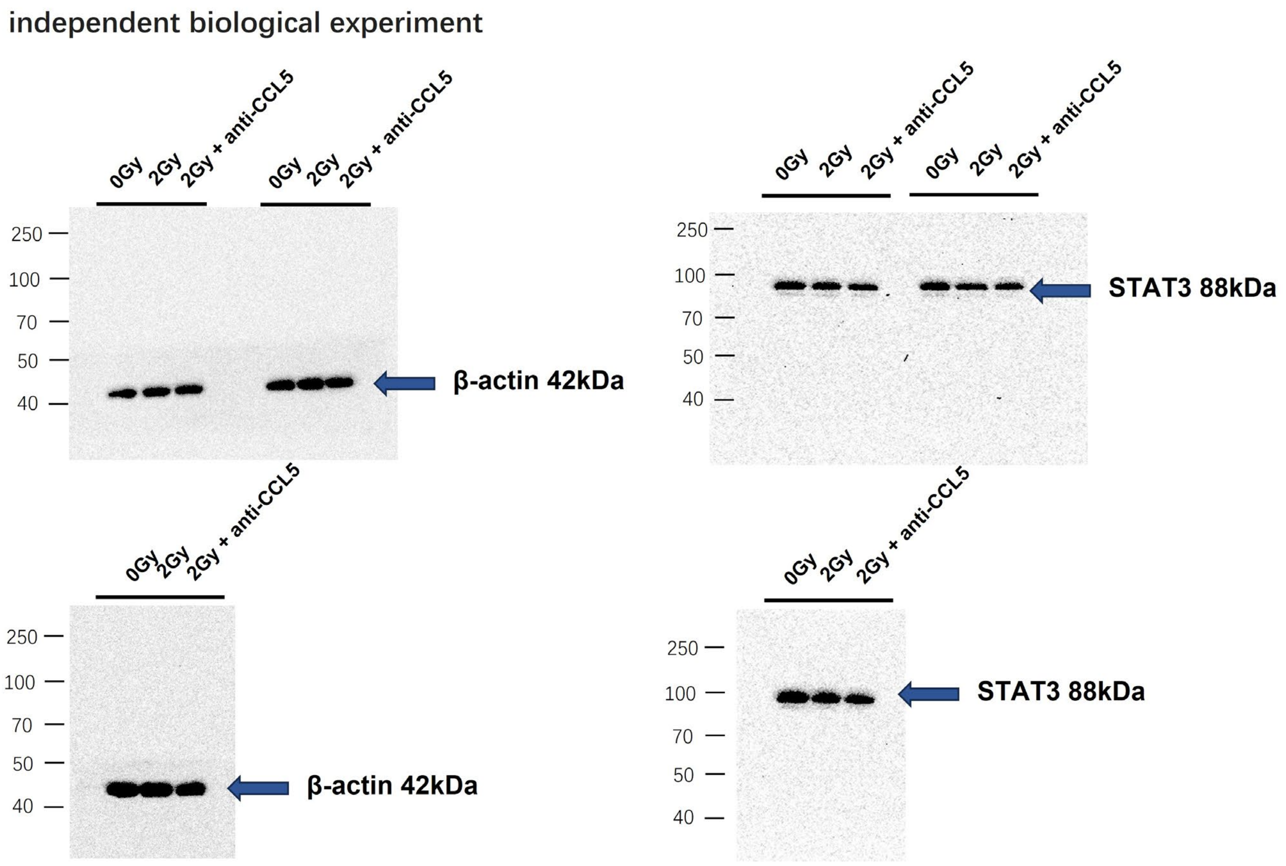

Figure 6A. Original blots of β-actin proteins.

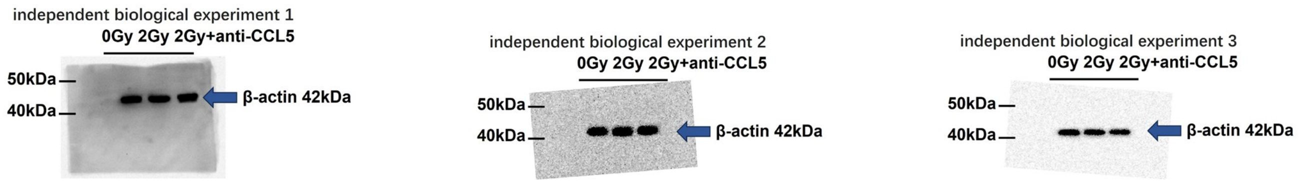

Figure 6A. Original blots of p-JAK1 proteins.

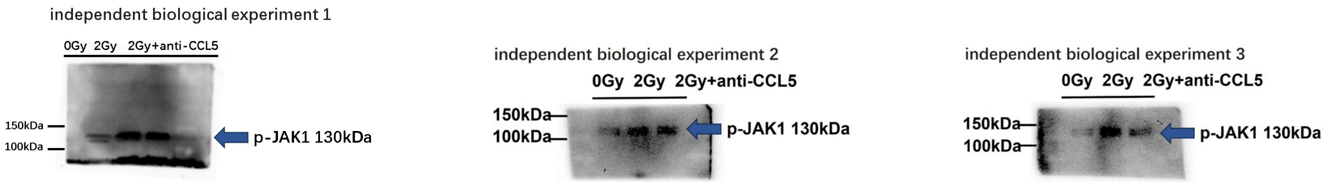

Figure 6B. Original blots of β-actin proteins.

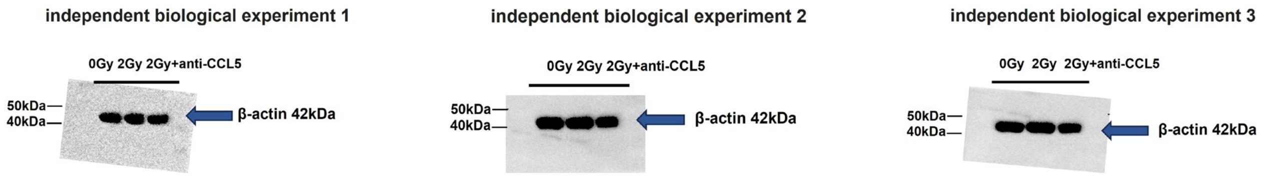

Figure 6B. Original blots of RANKL proteins.

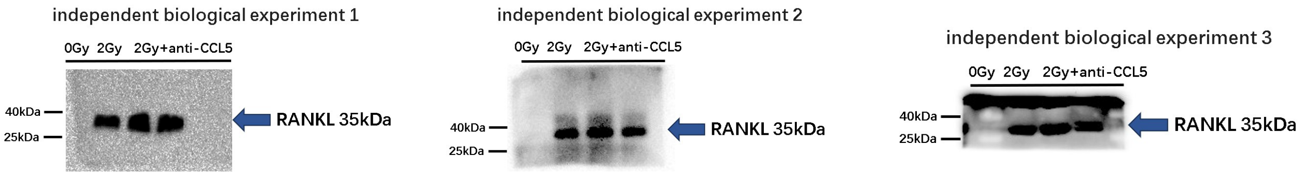

Figure 6B. Original blots of β-actin proteins.

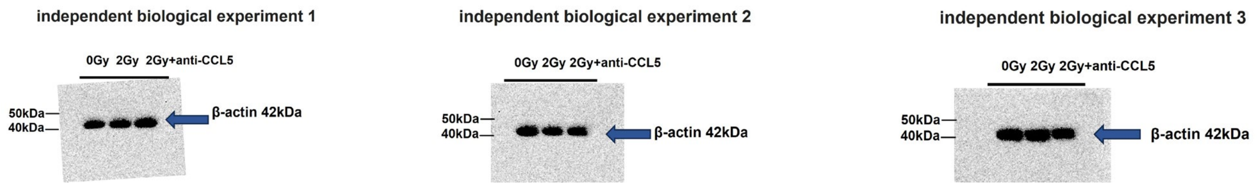

Figure 6B. Original blots of OPG proteins.

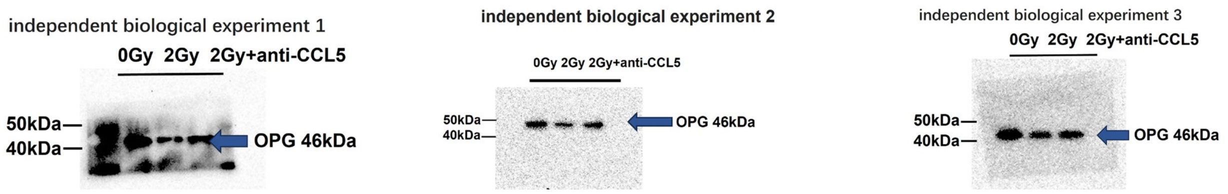

Supplement: Supplementary file 1 [file ijms-24-16168-s001.zip › Supplementary File S1.pdf]
