# Supplementary material for: C-C Motif Chemokine Ligand 5 (CCL5) Promotes Irradiation-Evoked Osteoclastogenesis
Source: Int J Mol Sci. 2023 Nov 10;24(22):16168. doi: 10.3390/ijms242216168 (PMC10671276; doi:10.3390/ijms242216168)
Supplement: Supplementary file 1 [file ijms-24-16168-s001.zip › Supplementary File S2.pdf]

C-C Motif Chemokine Ligand 5 (CCL5) Promotes Irradiation-Evoked Osteoclastogenesis

Jing Wang, Fanyu Zhao, Linshan Xu, Jianping Wang, Jianglong Zhai, Li Ren And Guoying Zhu*

Department of Radiological Hygiene, Institute of Radiation Medicine, Fudan University, 2094 Xietu Road, Shanghai 200032, China;
* Correspondence: zhugy@shmu.edu.cn; Tel.: +86-21-64049847

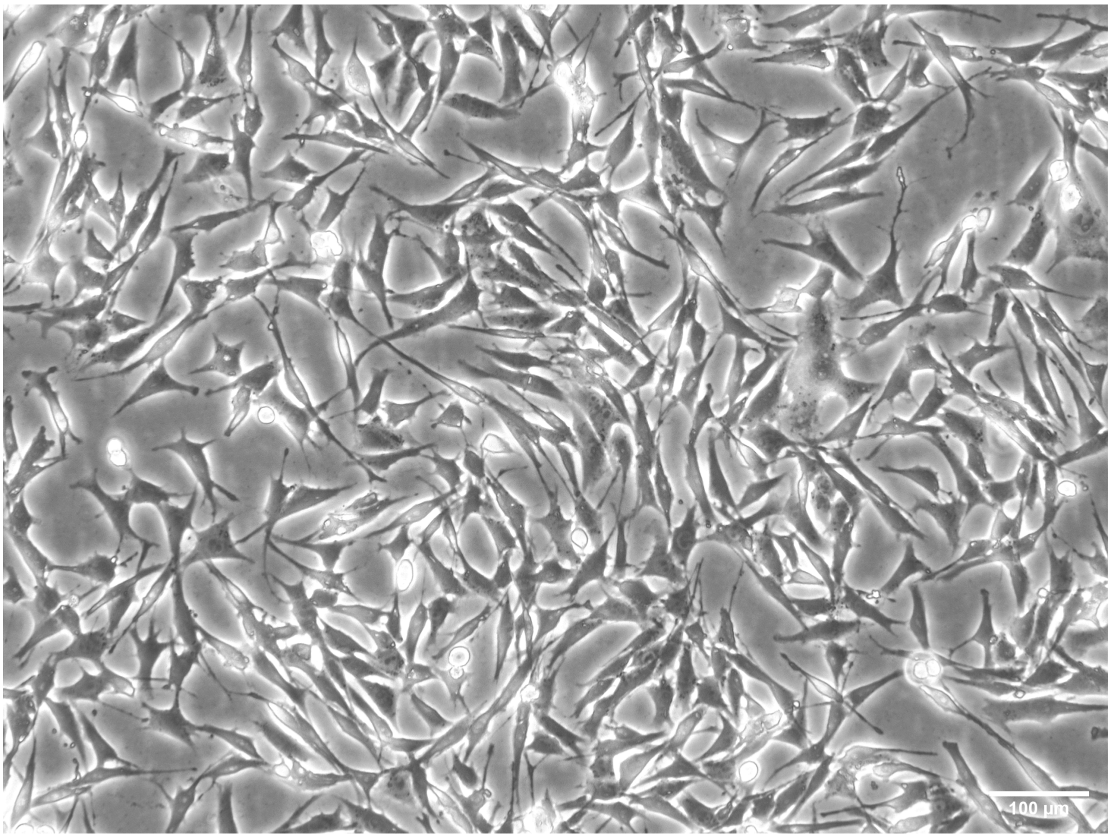

Figure S1. The original image of primary OCY morphology by LM; Magnification = x100.

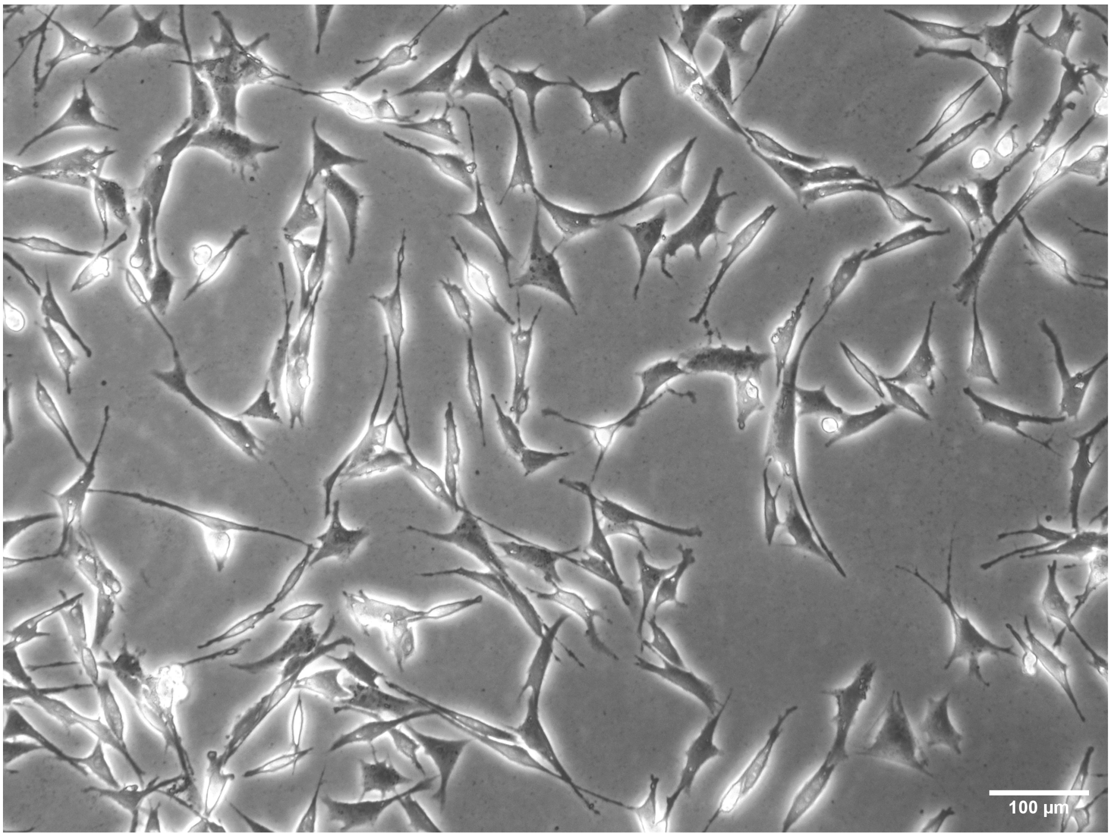

Figure S2. The original image of primary OCY morphology by LM at 3 days post-irradiation; Magnification = x100.

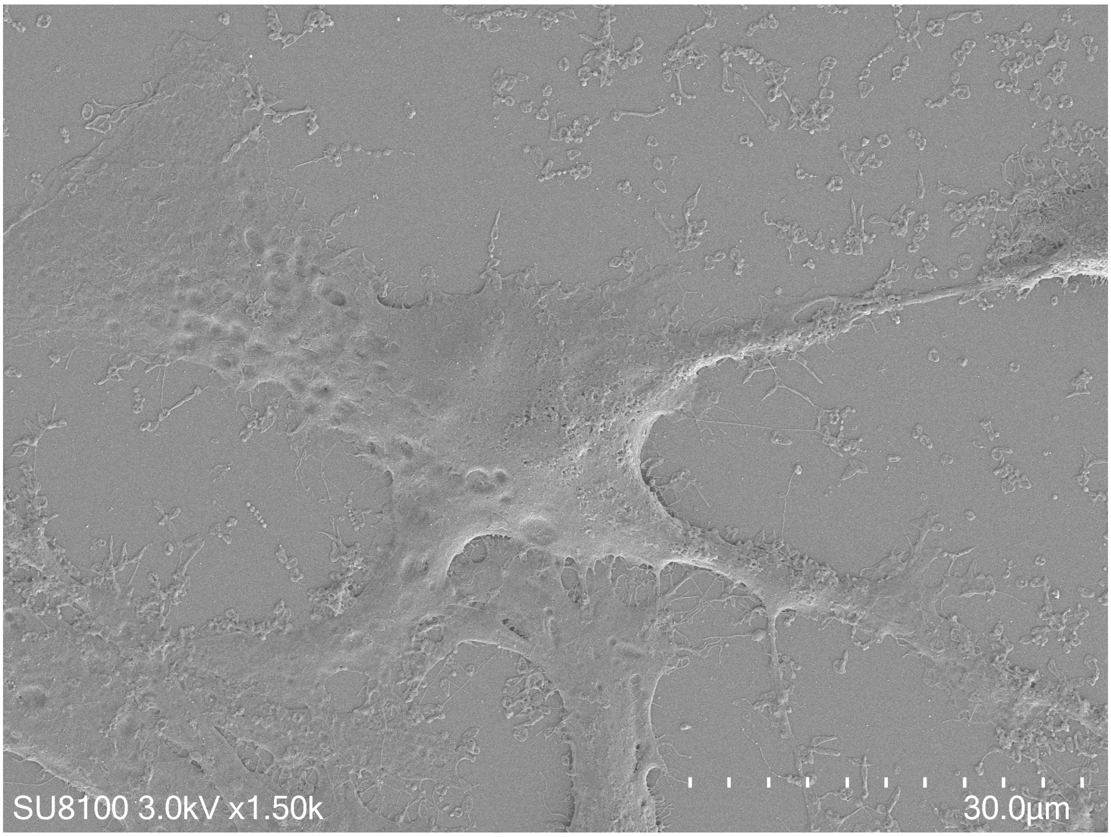

Figure S3. The original image of primary OCY morphology by SEM; Magnification = x1.5k.

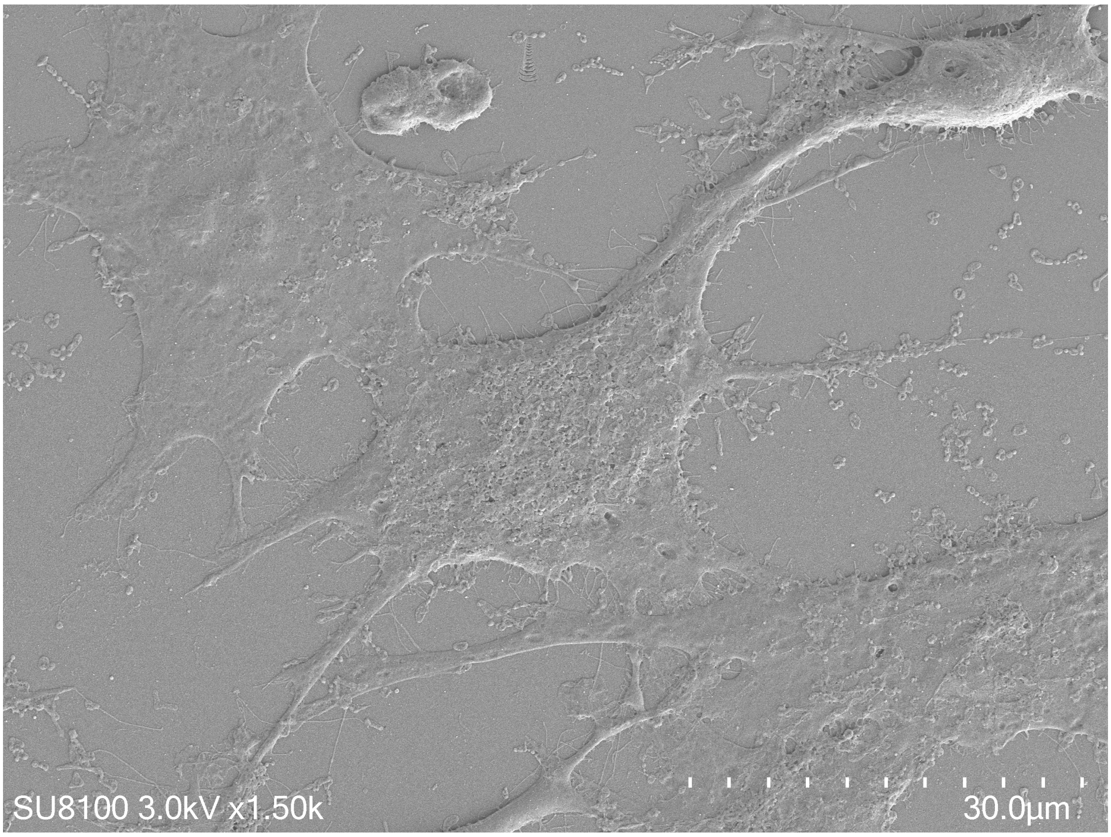

Figure S4. The original image of irradiated OCY morphology by SEM; Magnification = x1.5k.

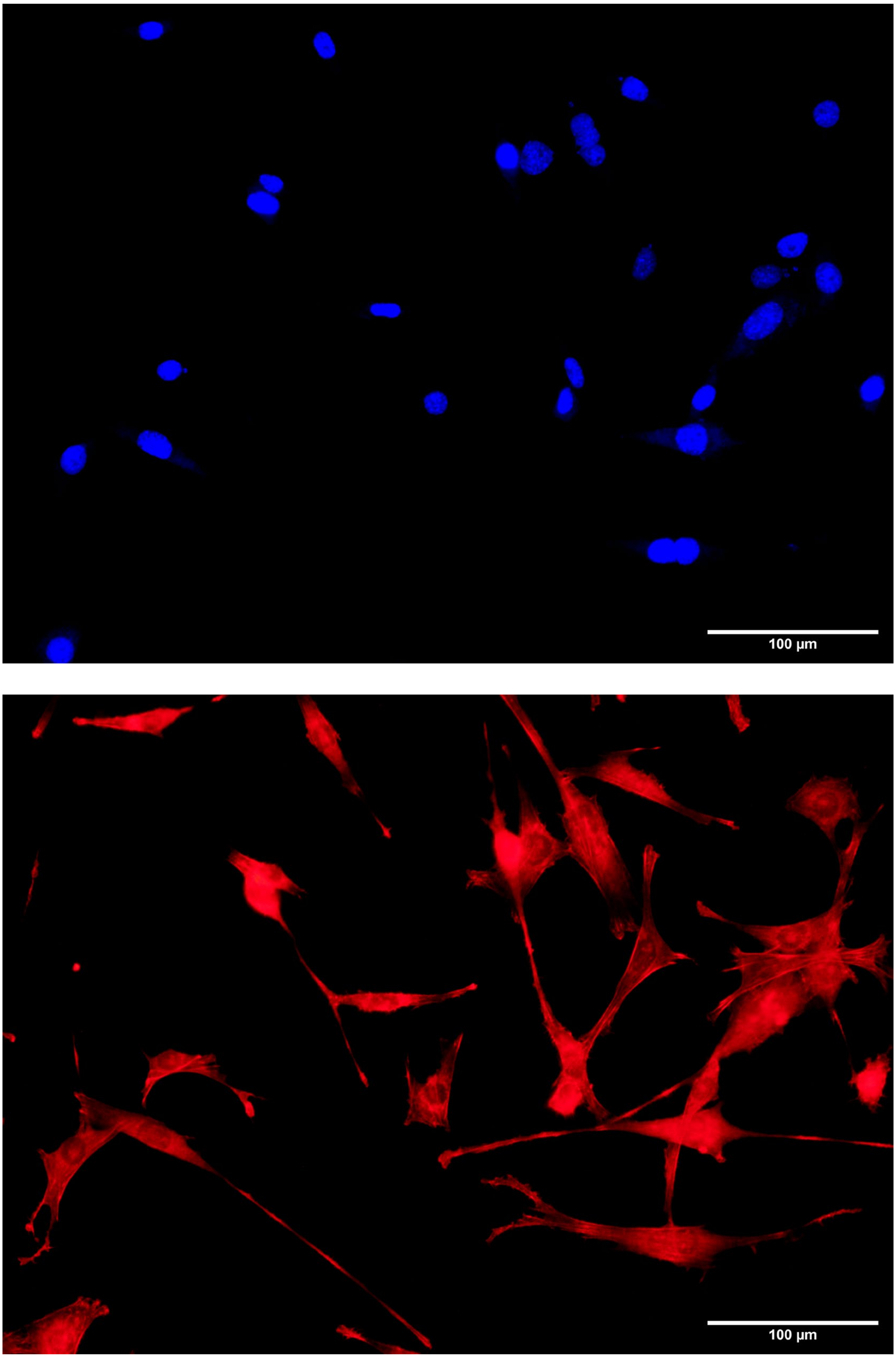

Figure S5. Immunofluorescence staining of OCYs using phalloidin-AlexaFluor488 to visualize the typical dendrite-like synapse (shown in red) and DAPI fluorescence to visualize the nuclei (shown in blue); Magnification = x200.

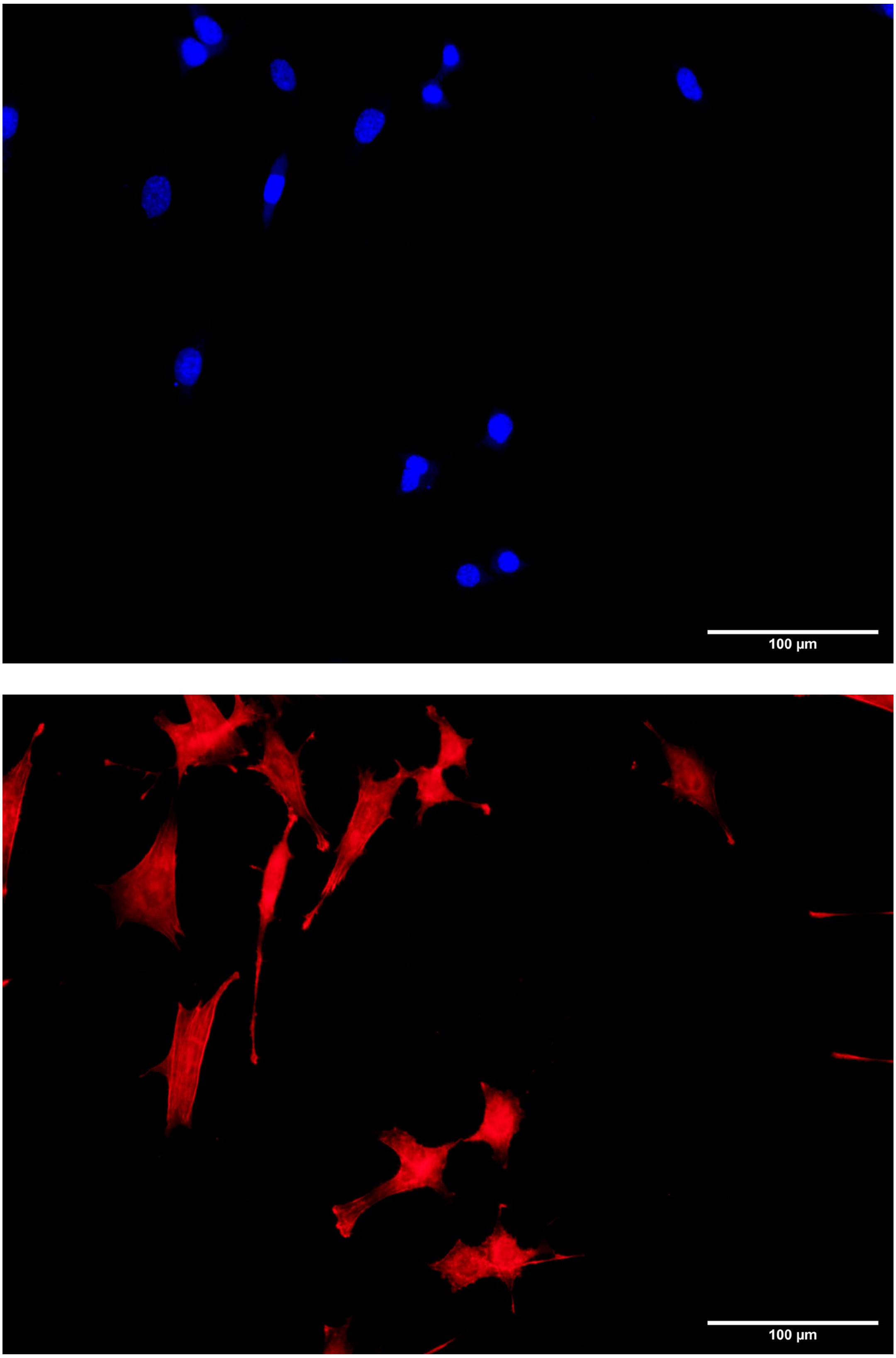

Figure S6. Immunofluorescence staining of irradiated OCYs, using phalloidin-AlexaFluor488 to visualize the typical dendrite-like synapse (shown in red) and DAPI fluorescence to visualize the nuclei (shown in blue); Magnification = x200.

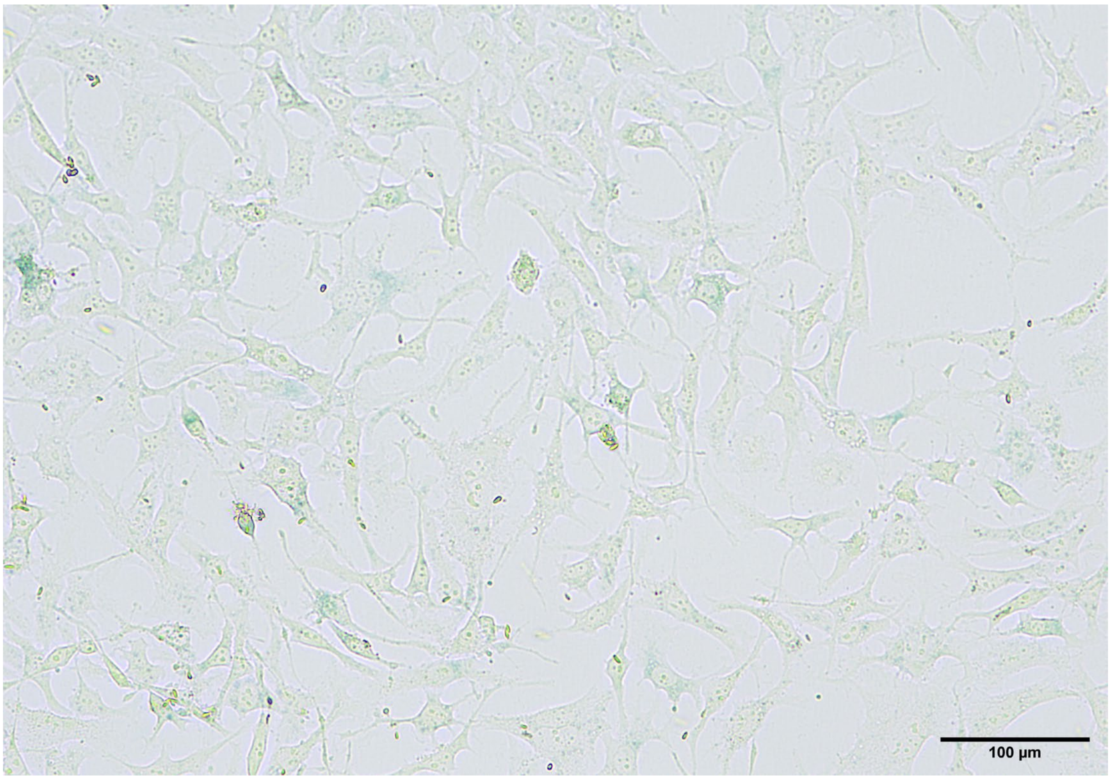

Figure S7. The original image of SA-β-gal staining in primary OCYs; Magnification = x100.

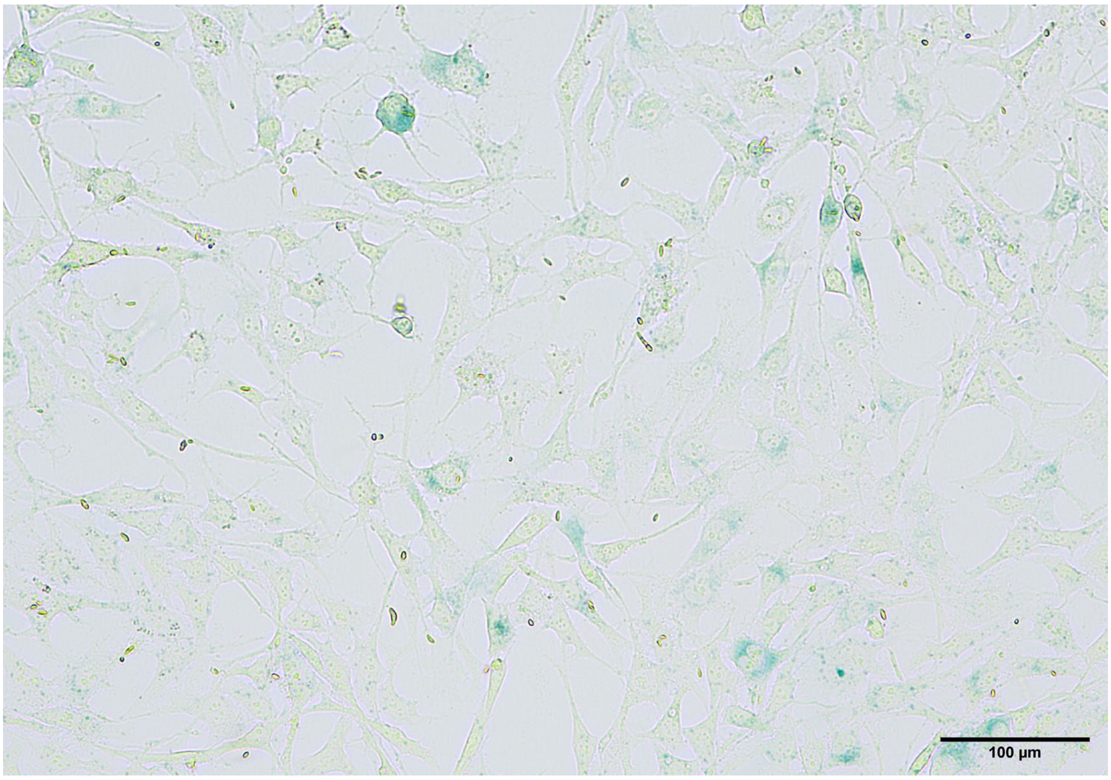

Figure S8. The original image of SA-β-gal staining in irradiated OCYs; Magnification = x100.

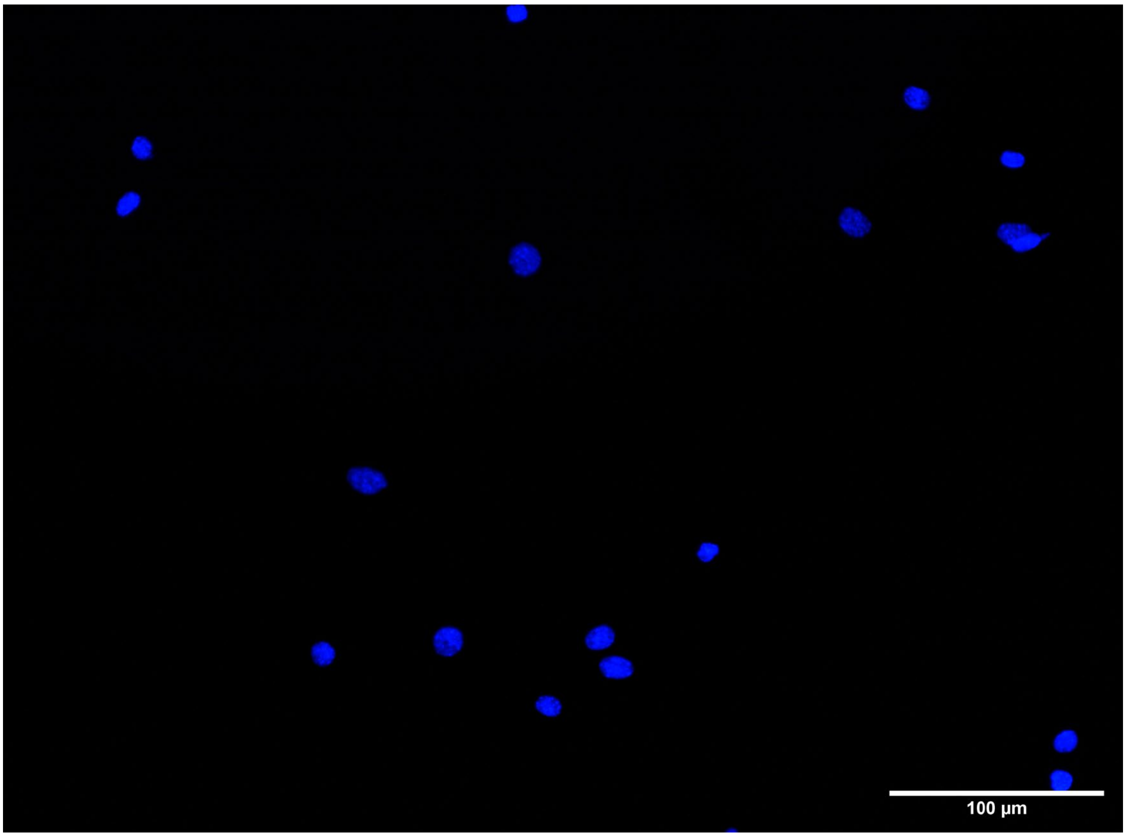

Figure S9. The original image of SAHF formation in OCYs nuclei by FM; Magnification = x200.

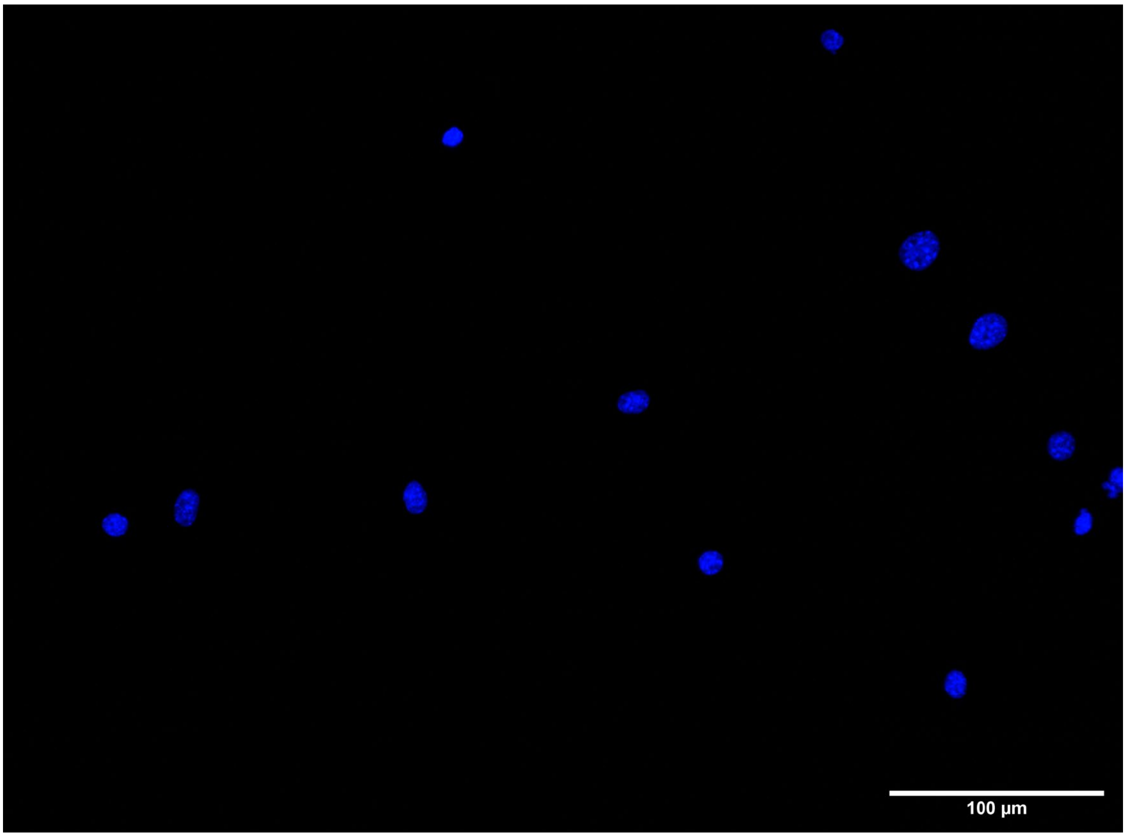

Figure S10. The original image of SAHF formation in the nucleus of irradiated OCYs by FM; Magnification = x200.

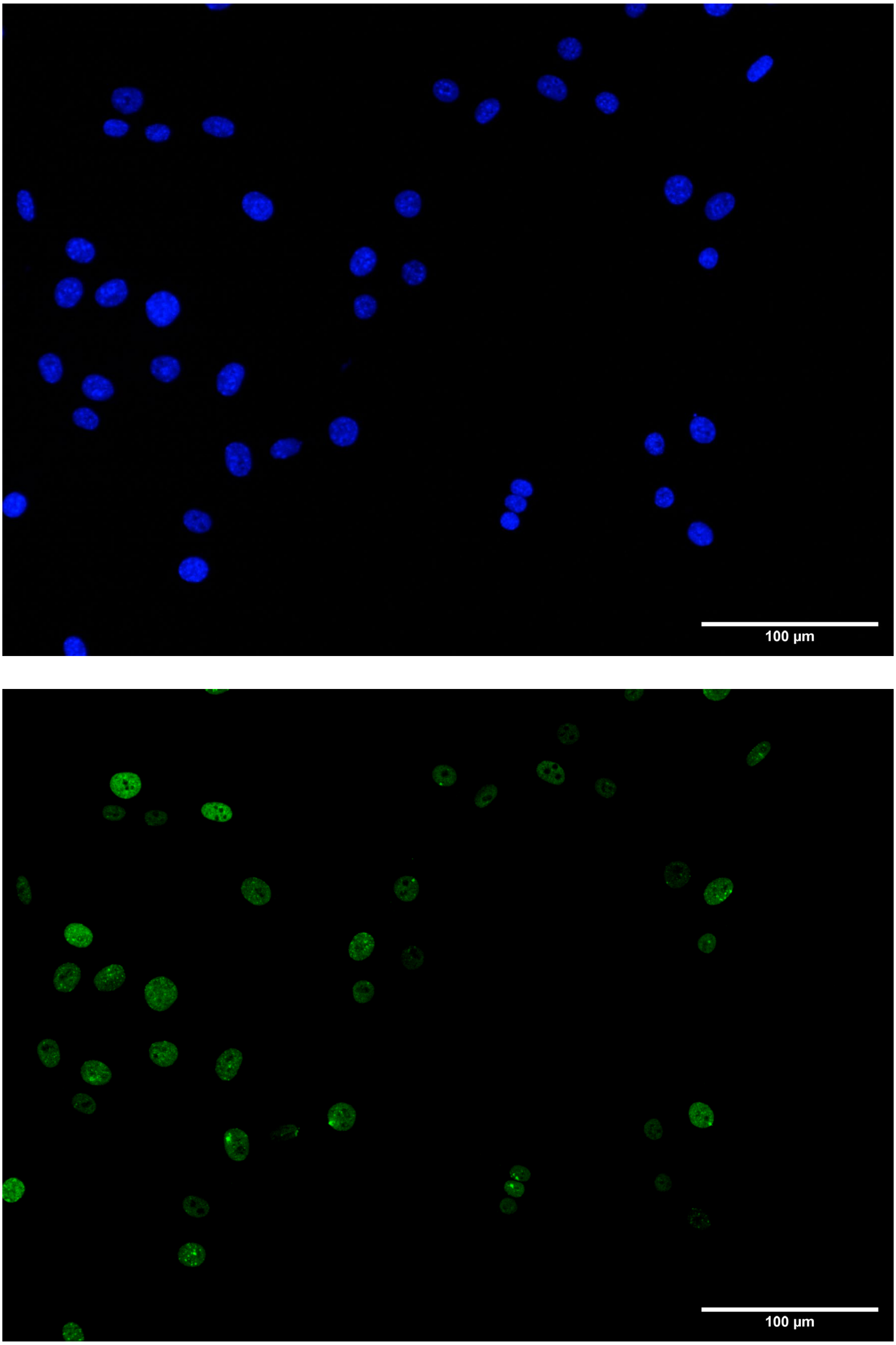

Figure S11. Immunofluorescence staining for γ- H2AX of OCYs: γ- H2AX (shown green) and DAPI (shown in blue); Magnification = x200.

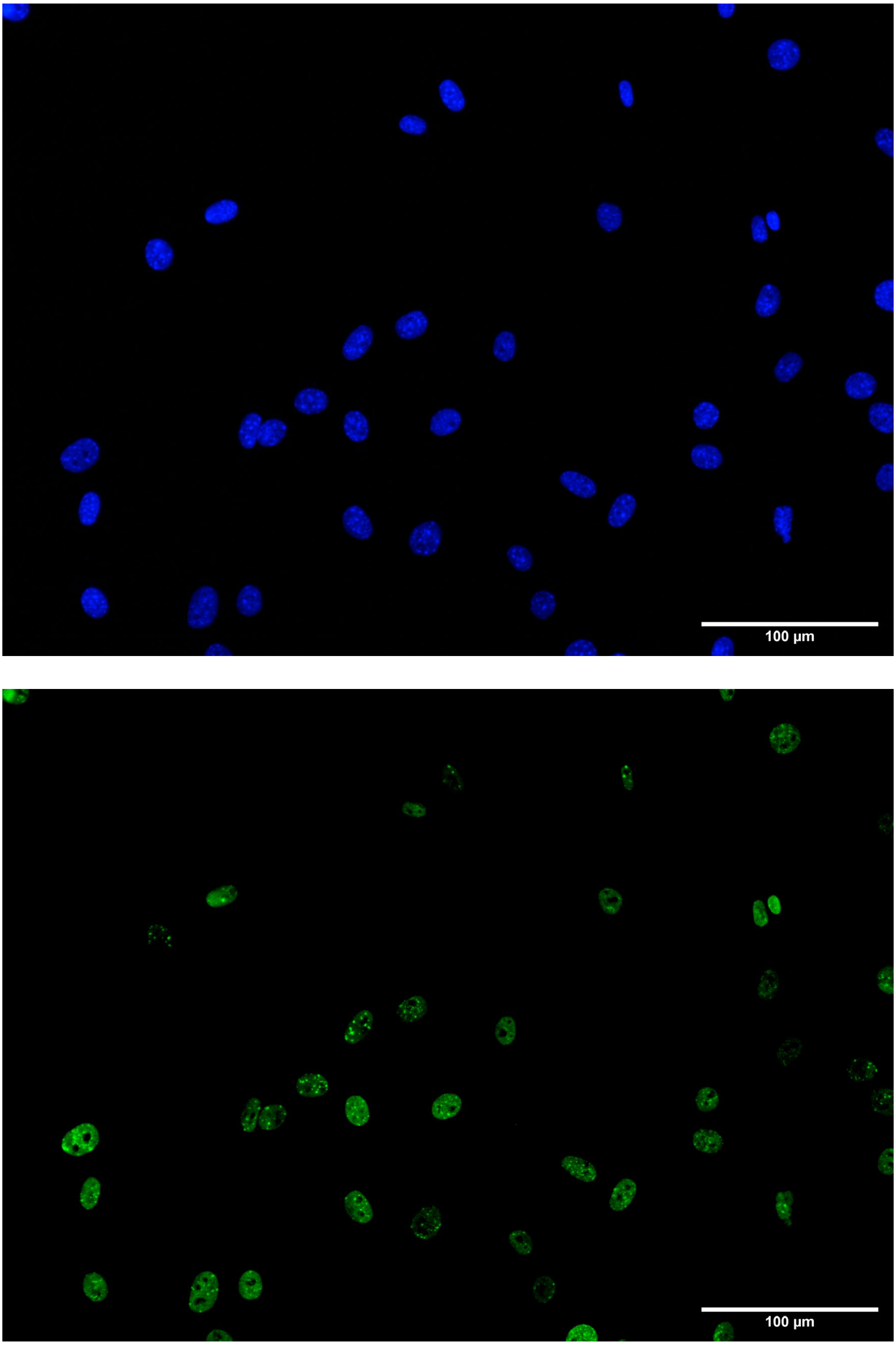

Figure S12. Immunofluorescence staining for γ- H2AX of irradiated OCYs: γ- H2AX (shown green) and DAPI (shown in blue); Magnification = x200.

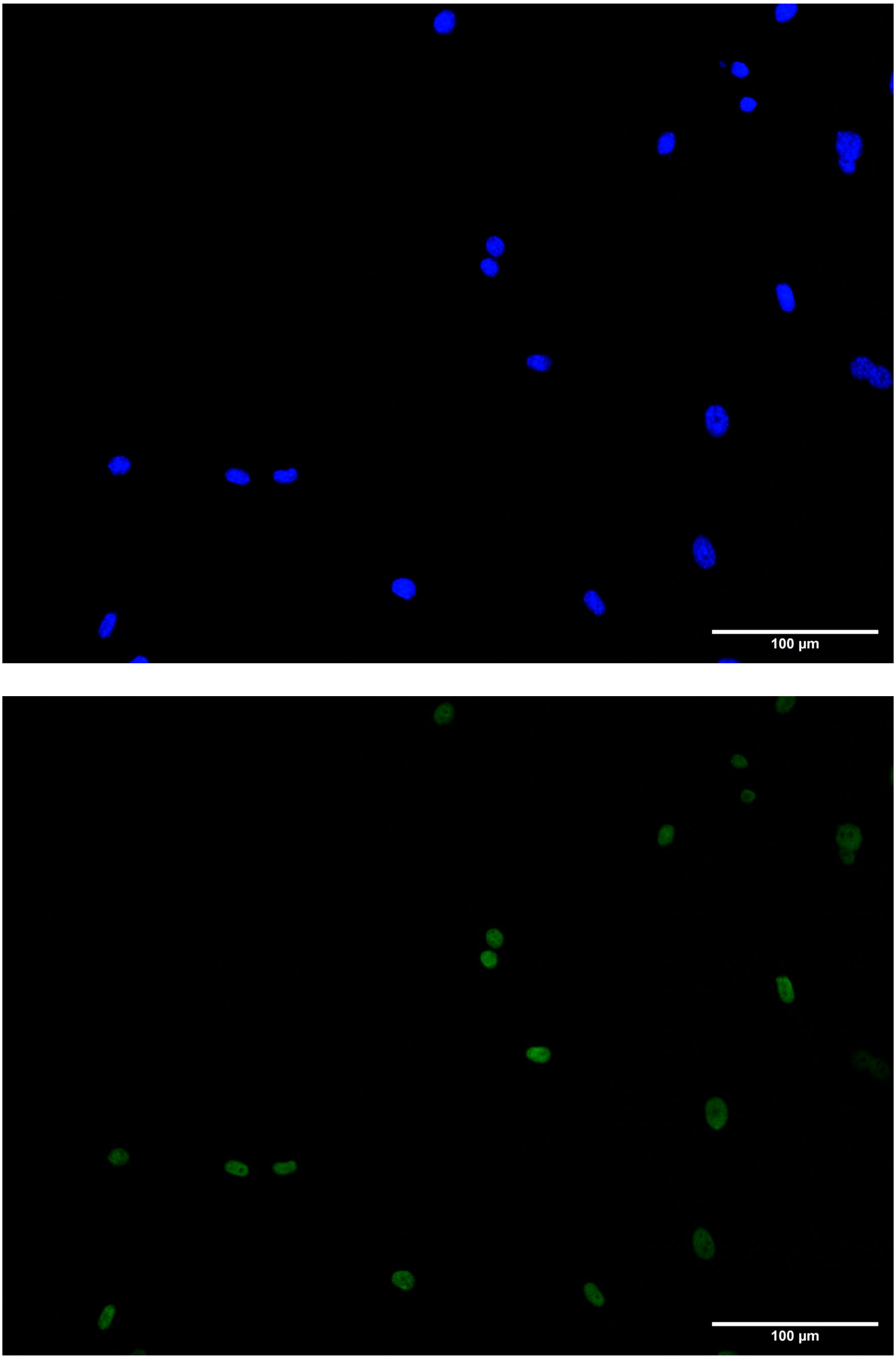

Figure S13. Immunofluorescence staining for CCL5 of OCYs: CCL5 (shown in green) and DAPI (shown in blue); Magnification = x200.

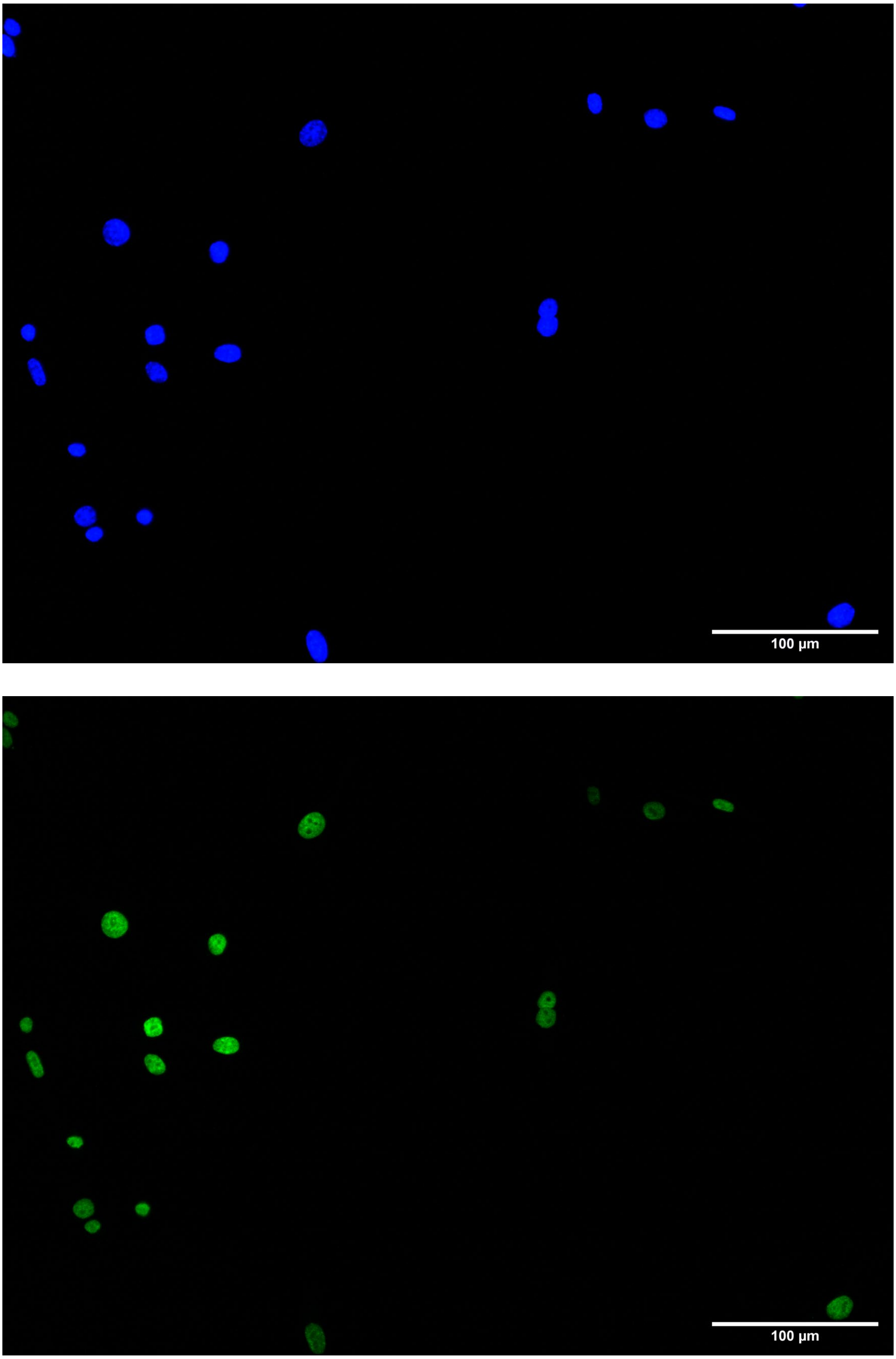

Figure S14. Immunofluorescence staining for CCL5 of irradiated OCYs: CCL5 (shown in green) and DAPI (shown in blue); Magnification = x200.

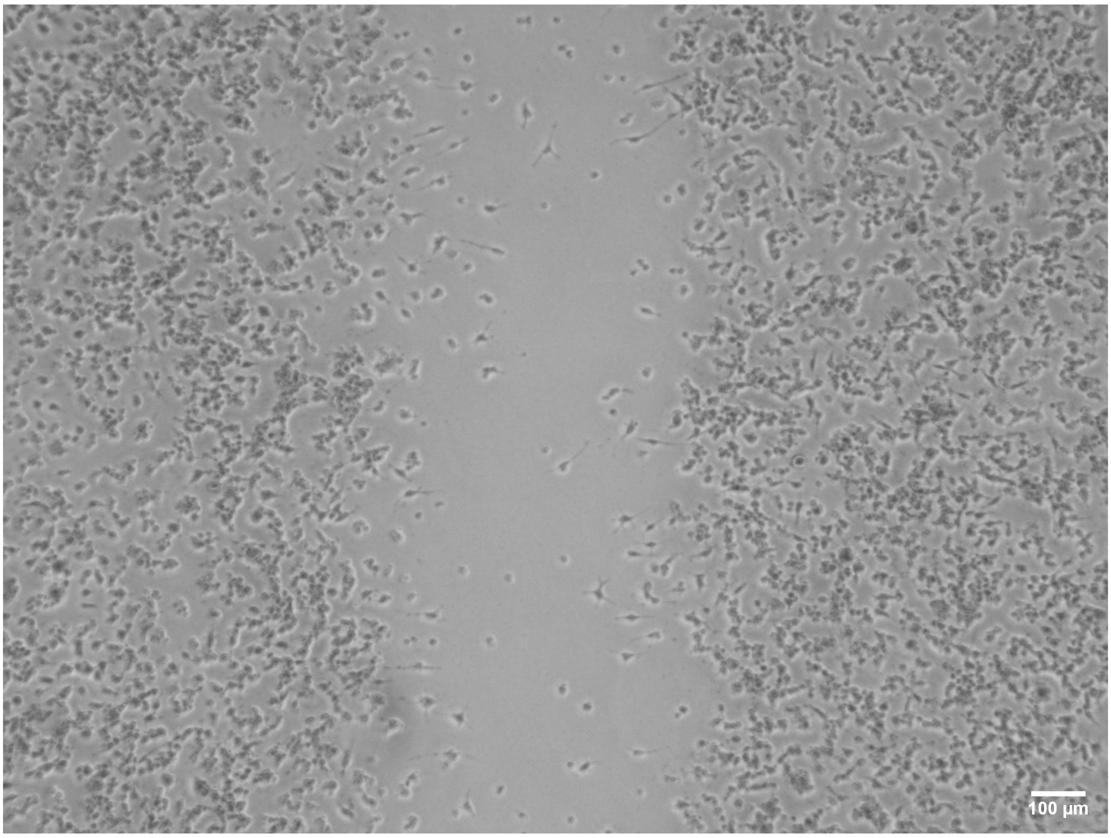

Figure S15. Changes in the migration rate of RAW264.7 precursor cells co-cultured with CM-0 Gy by wound-healing assay; Magnification = x20.

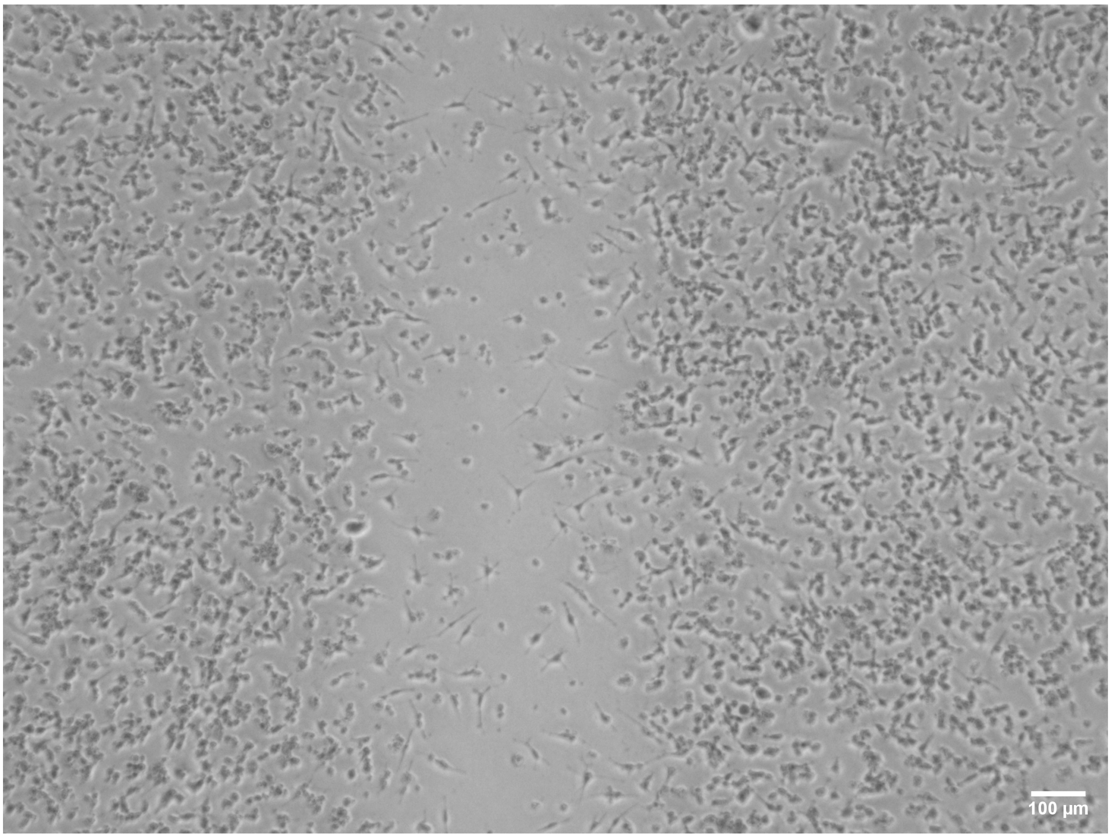

Figure S16. Changes in the migration rate of RAW264.7 precursor cells co-cultured with CM-2 Gy by wound-healing assay; Magnification = x20.

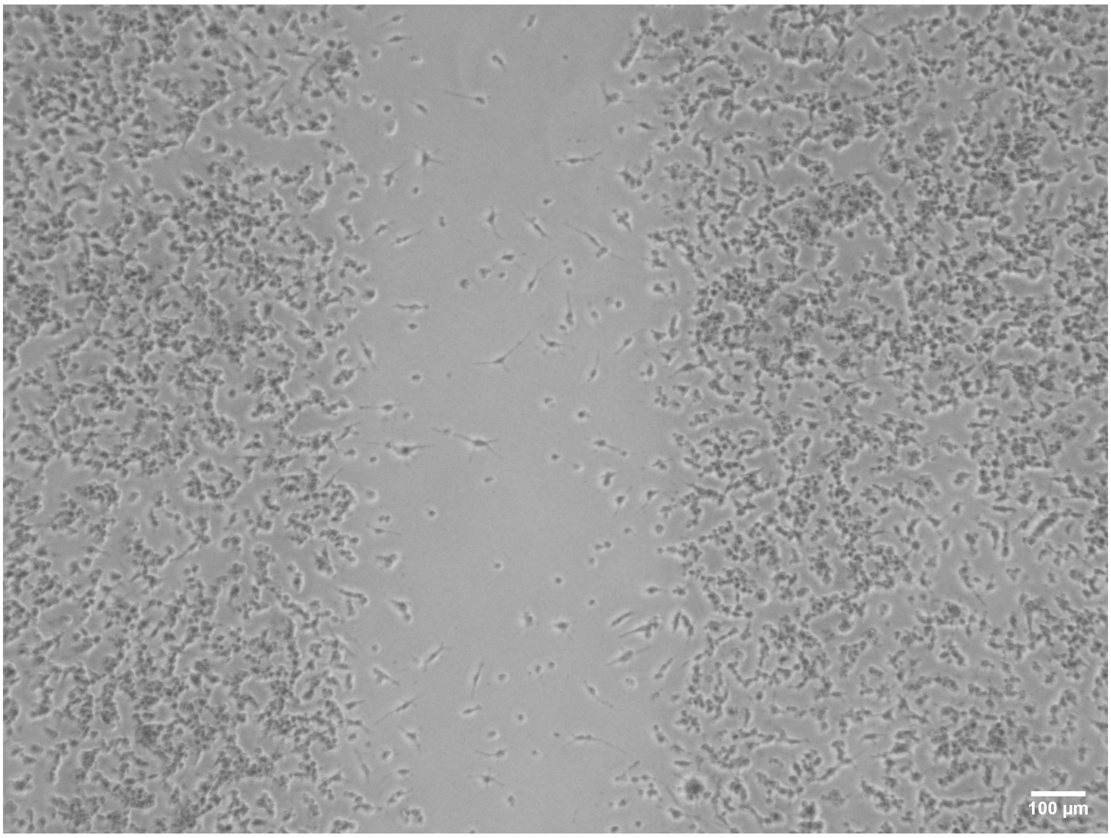

Figure S17. Changes in the migration rate of RAW264.7 precursor cells co-cultured with CM-2 Gy + anti-CCL5 by wound-healing assay; Magnification = x20.

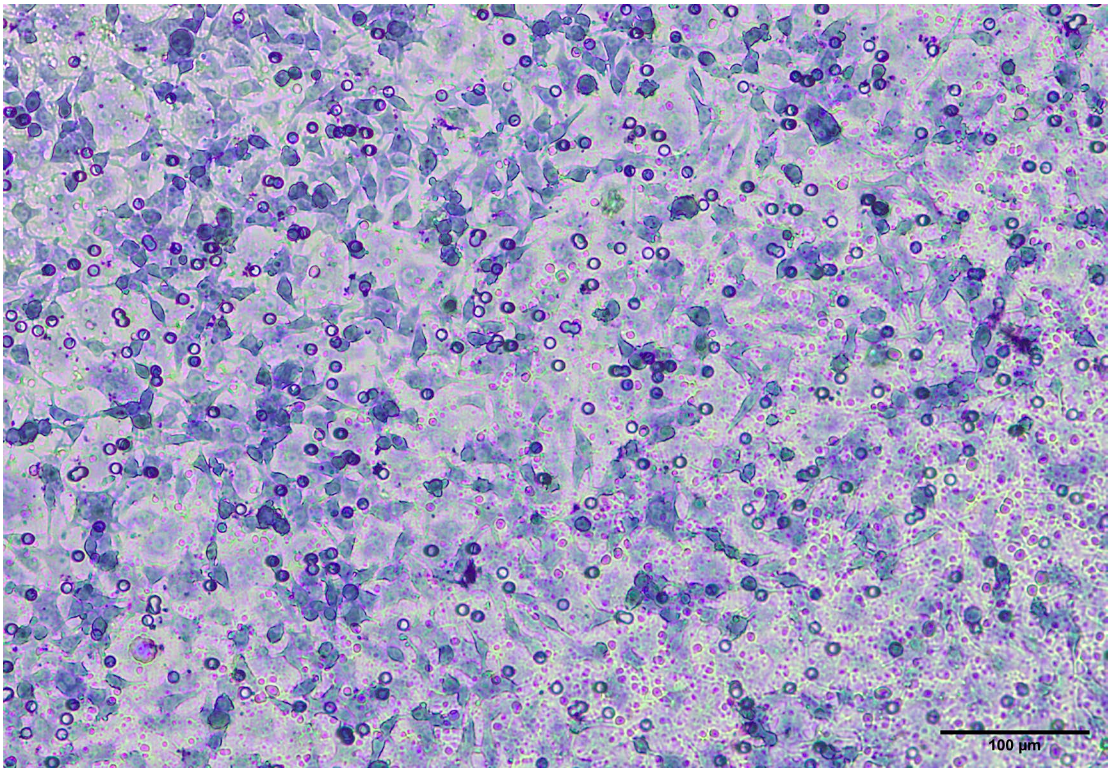

Figure S18. Changes in the migration numbers of RAW264.7 precursor cells co-cultured with CM-0 Gy by trans-well migration assay; Magnification = x100.

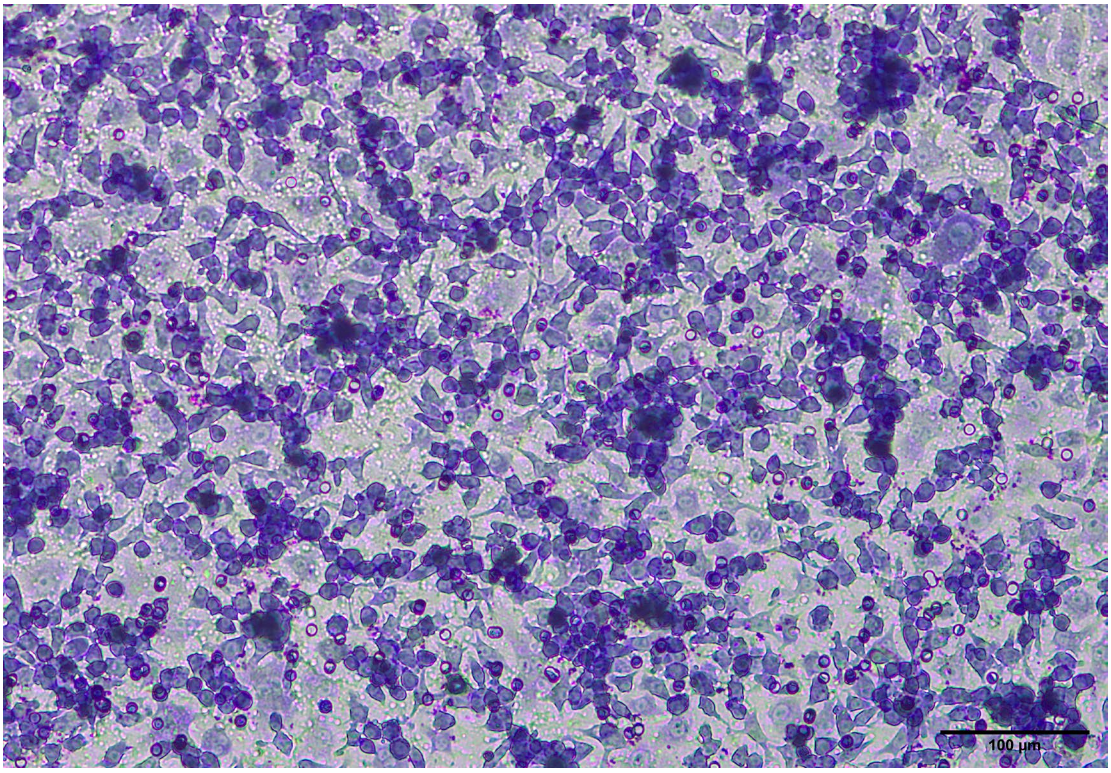

Figure S19. Changes in the migration numbers of RAW264.7 precursor cells co-cultured with CM-2 Gy by trans-well migration assay; Magnification = x100.

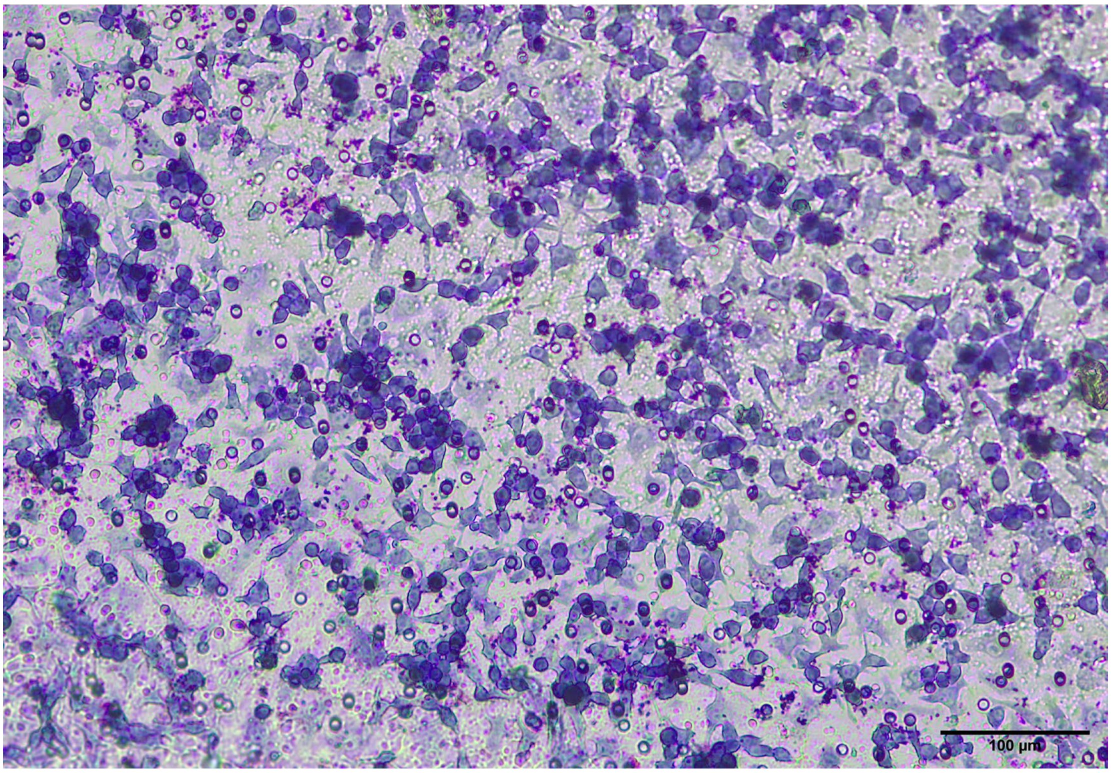

Figure S20. Changes in the migration numbers of RAW264.7 precursor cells co-cultured with CM-2 Gy + anti-CCL5 by trans-well migration assay; Magnification = x100.

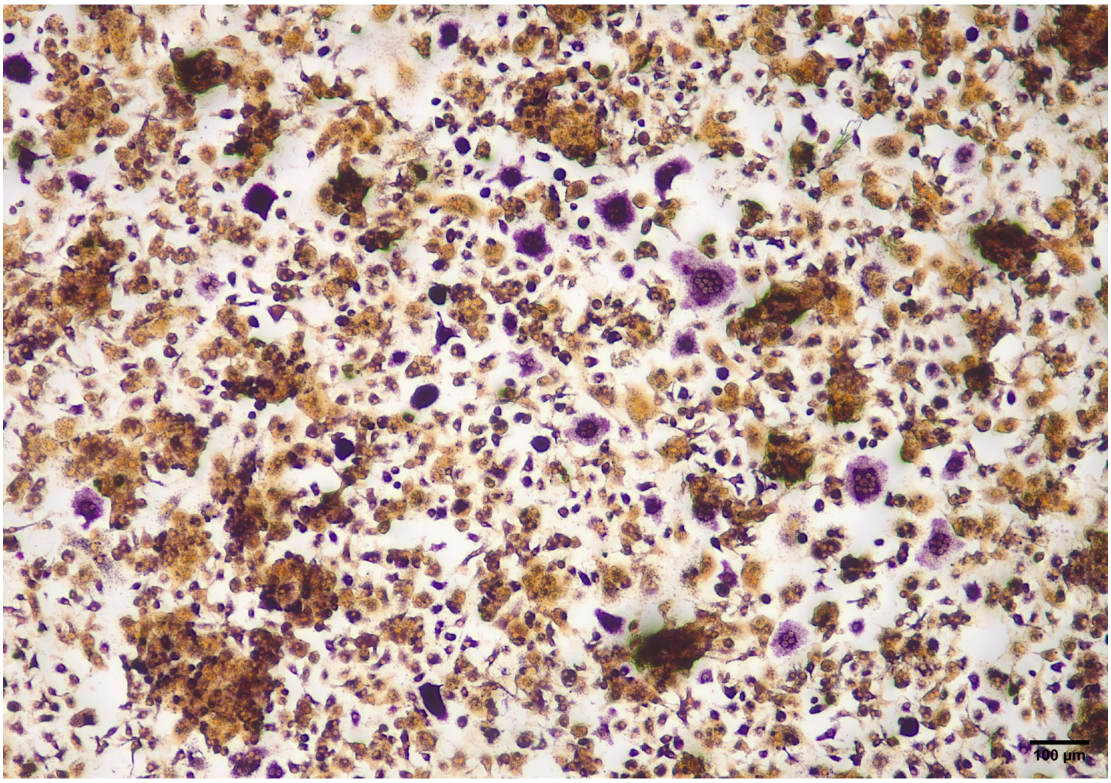

Figure S21. Changes in the TRAP+ area and numbers of OCs co-cultured with CM-0 Gy by TRAP staining; Magnification = x40.

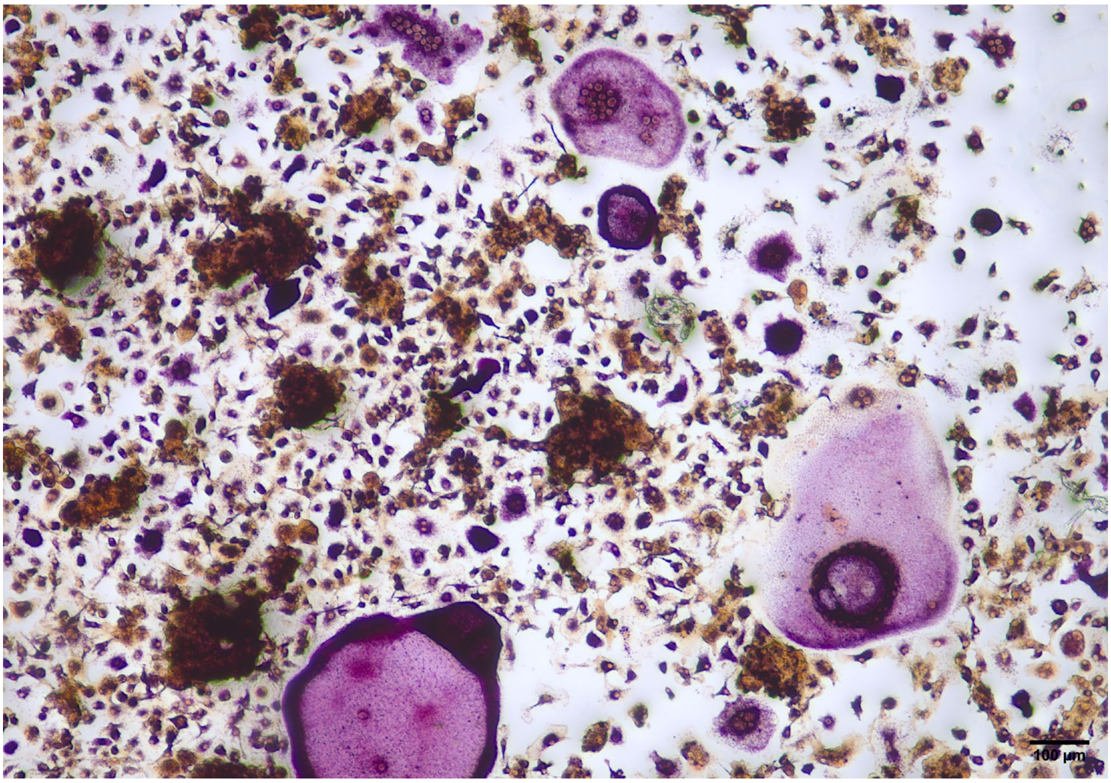

Figure S22. Changes in the TRAP+ area and numbers of OCs co-cultured with CM-2 Gy by TRAP staining; Magnification = x40.

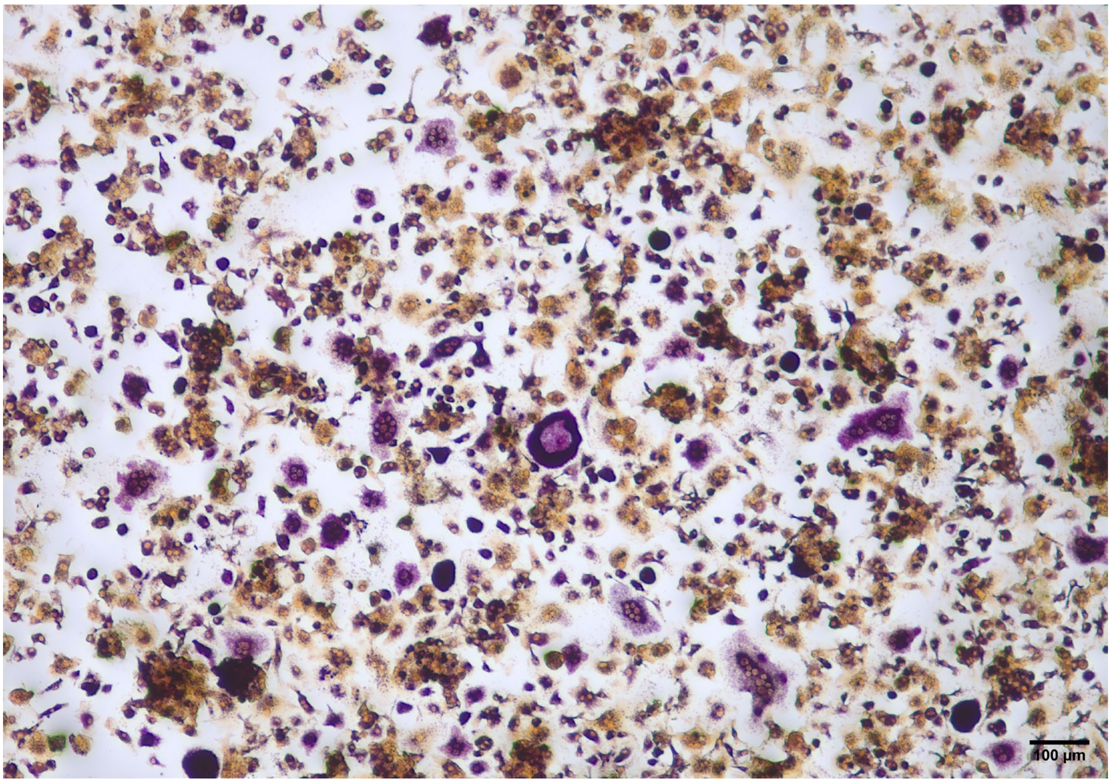

Figure S23. Changes in the TRAP+ area and numbers of OCs co-cultured with CM-2 Gy + anti-CCL5 by TRAP staining; Magnification = x40.

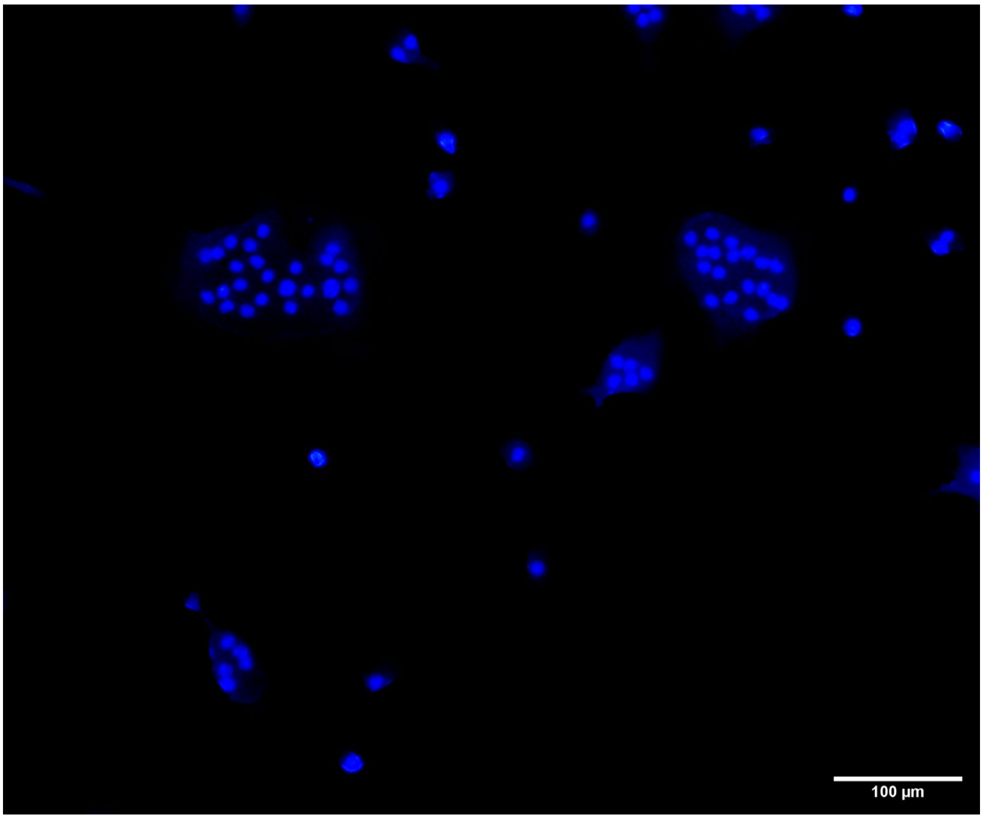

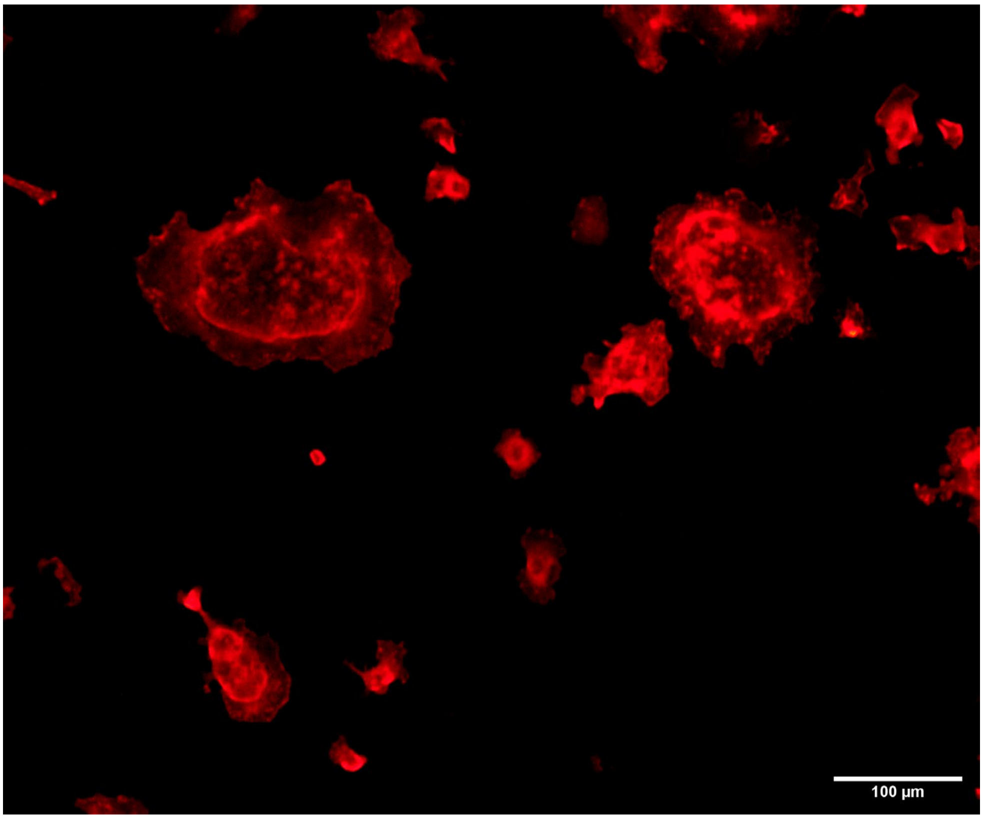

Figure S24. Immunofluorescence staining of the OC co-cultured with CM-0 Gy using phalloidin-AlexaFluor488 to visualize the typical OC actin rings (shown in red) and DAPI fluorescence to visualize the nuclei (shown in blue); Magnification = x100.

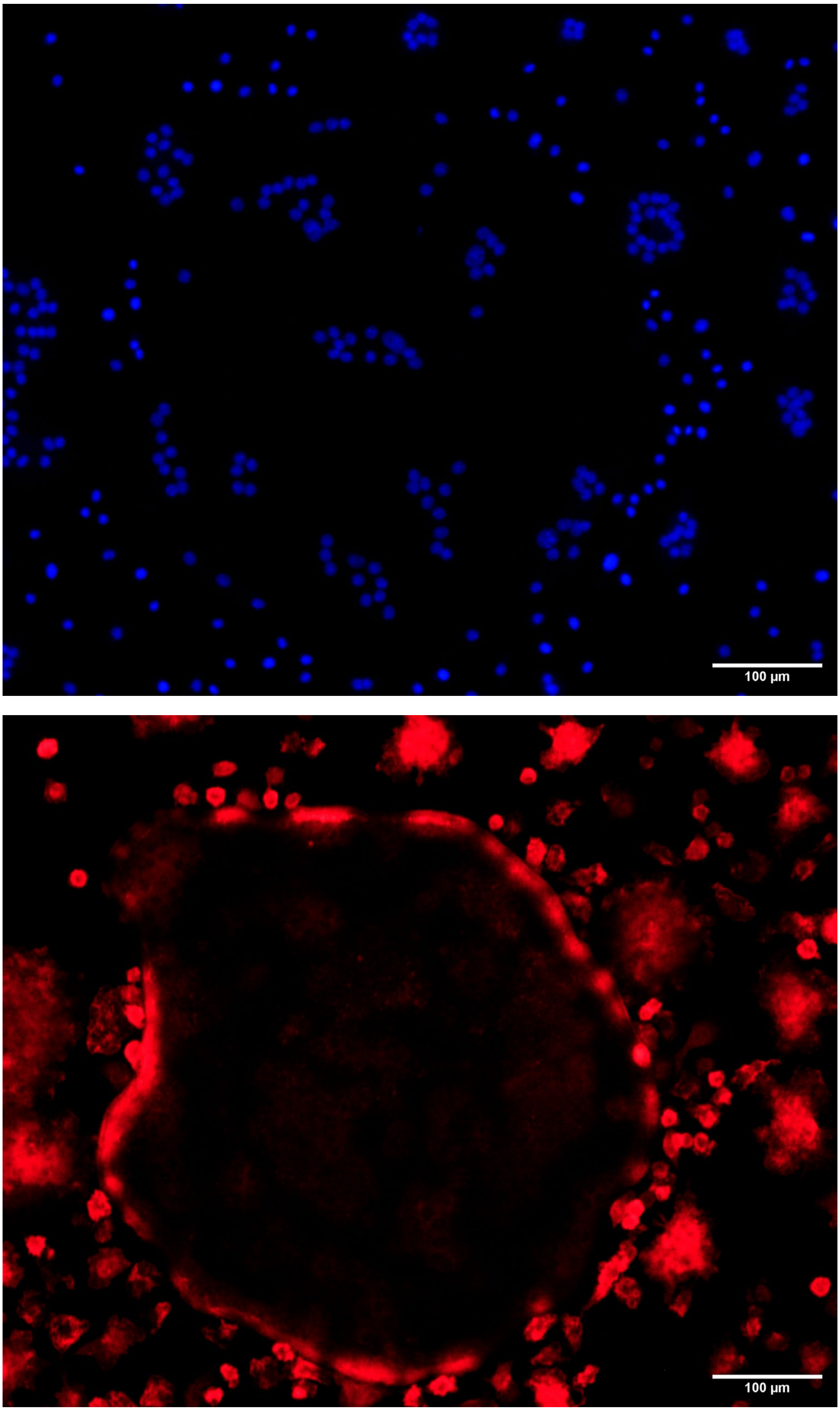

Figure S25. Immunofluorescence staining of the OC co-cultured with CM-2 Gy using phalloidin-AlexaFluor488 to visualize the typical OC actin rings (shown in red) and DAPI fluorescence to visualize the nuclei (shown in blue); Magnification = x100.

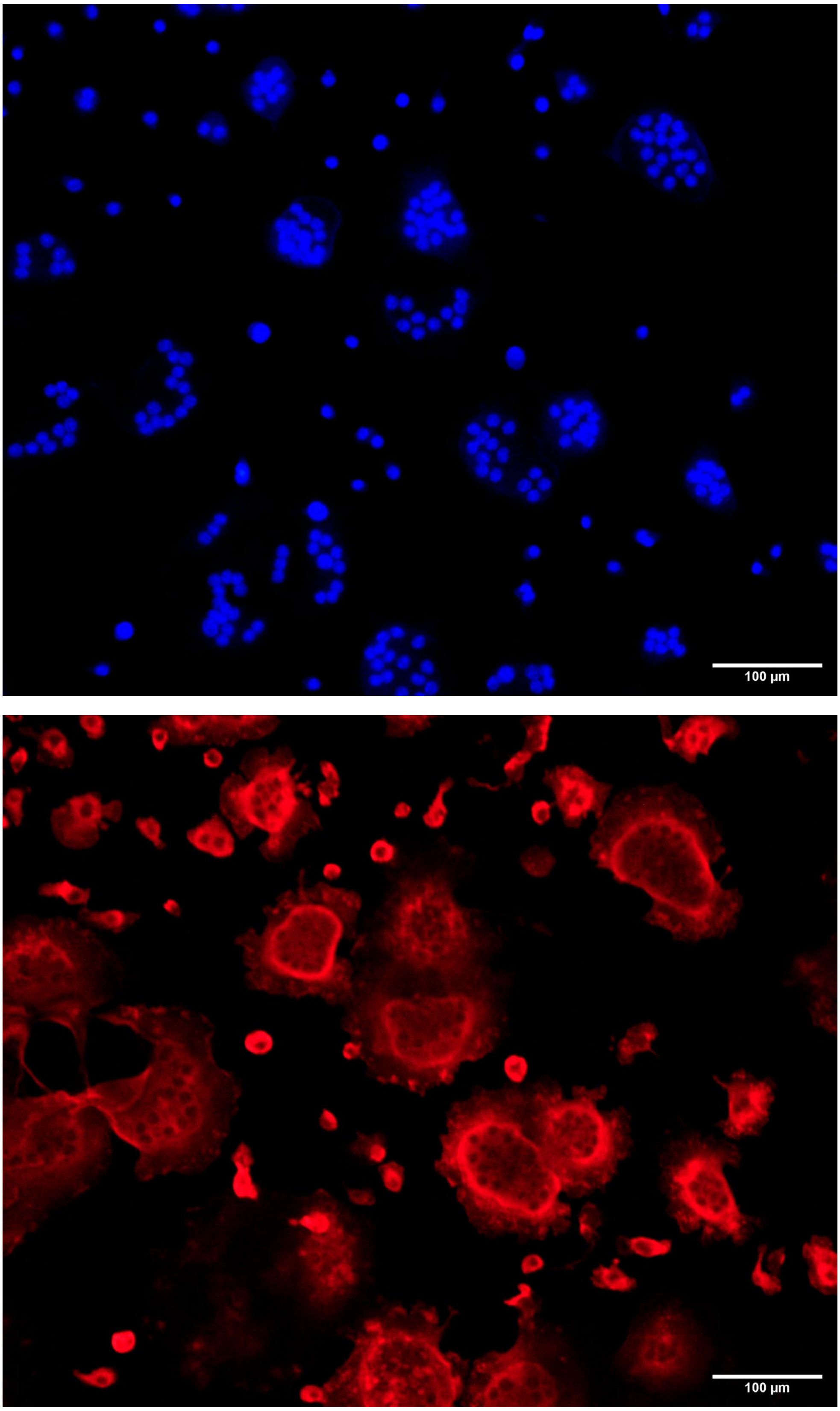

Figure S26. Immunofluorescence staining of the OC co-cultured with CM-2 Gy + anti-CCL5 using phalloidin-AlexaFluor488 to visualize the typical OC actin rings (shown in red) and DAPI fluorescence to visualize the nuclei (shown in blue); Magnification = x100.

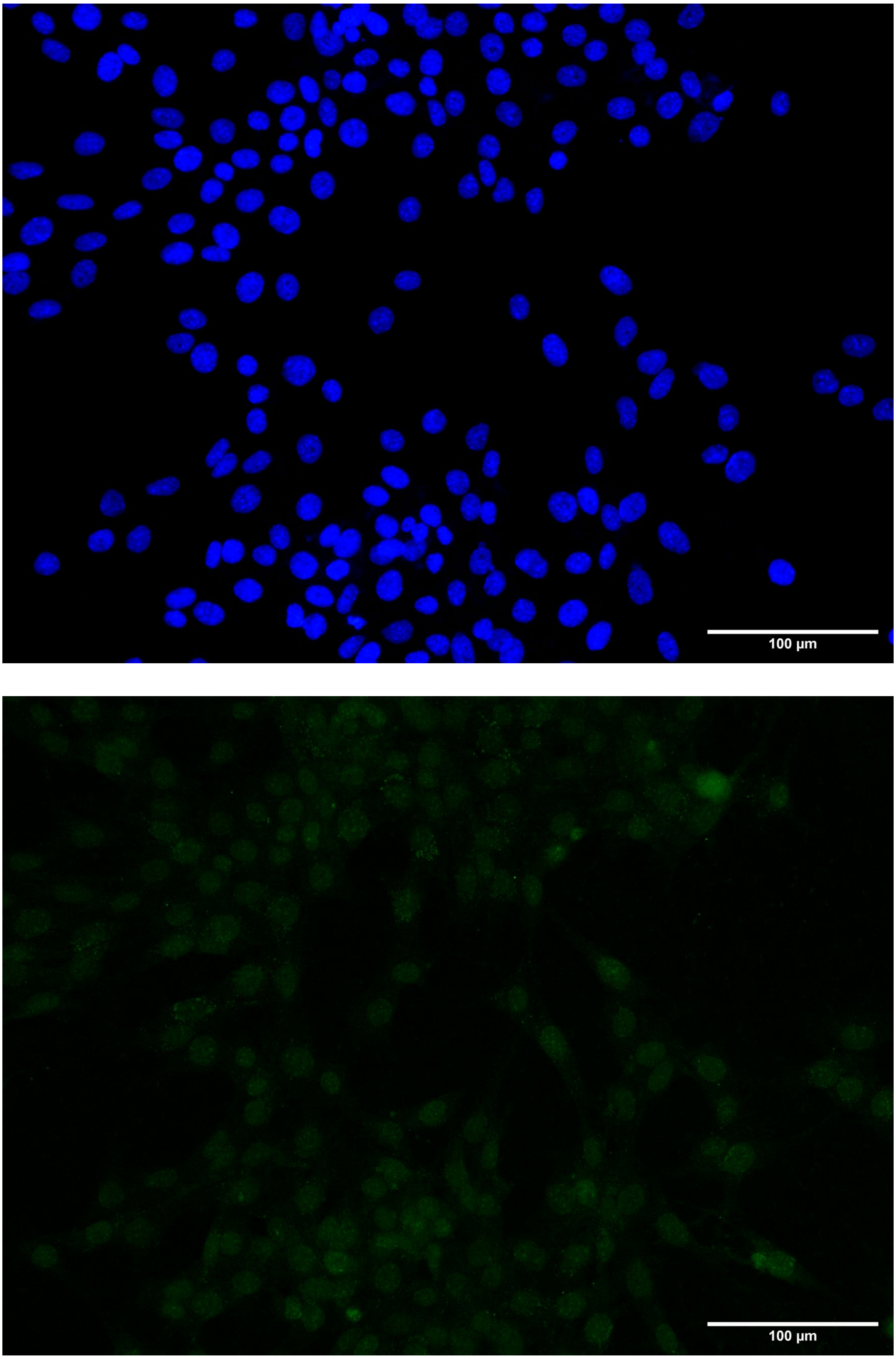

Figure S27. Immunofluorescence staining for p-STAT3 of OCYs: p-STAT3 (shown in green) and DAPI (shown in blue); Magnification = x200.

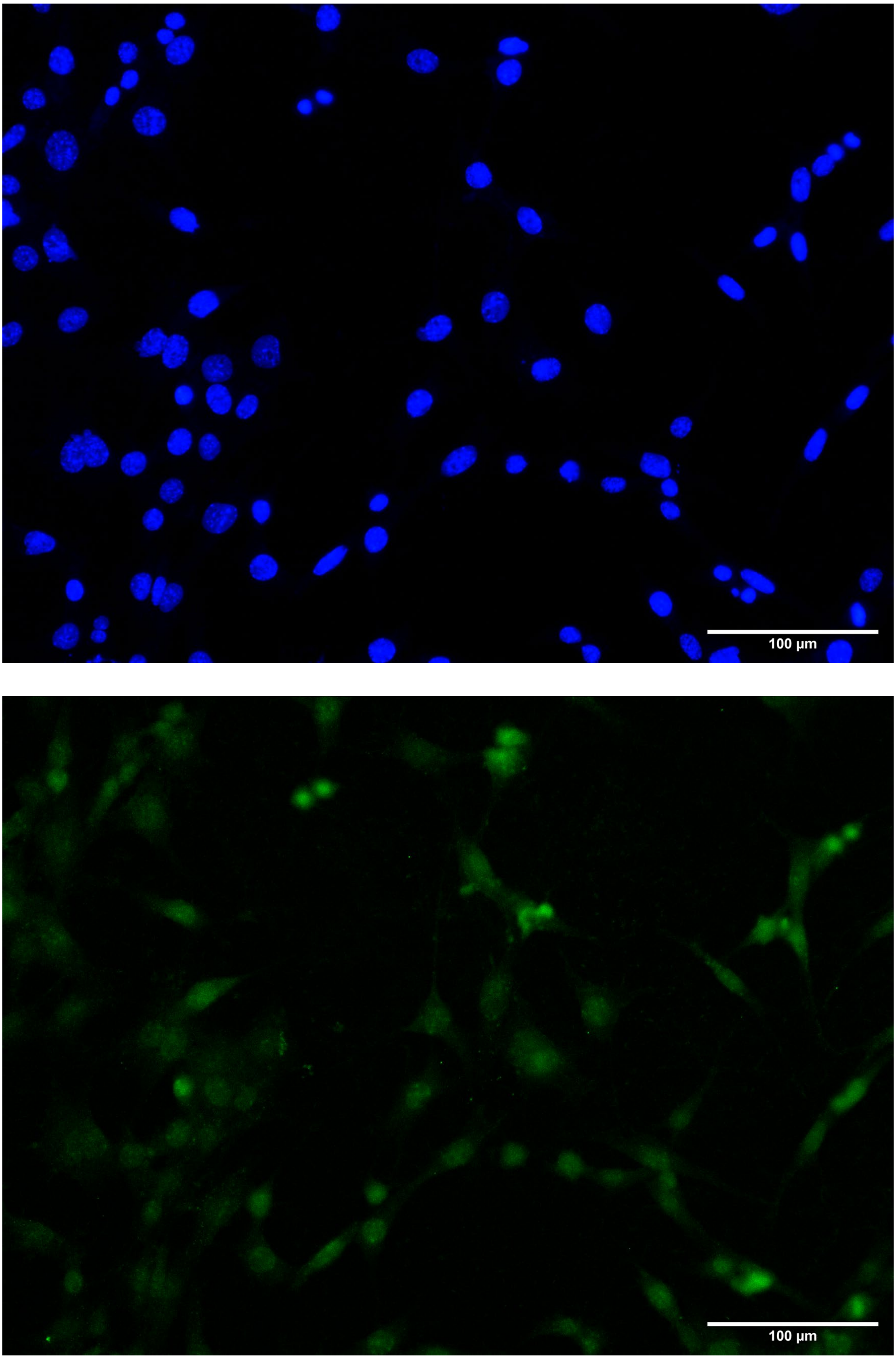

Figure S28. Immunofluorescence staining for p-STAT3 of irradiated OCYs: p-STAT3 (shown in green) and DAPI (shown in blue); Magnification = x200.

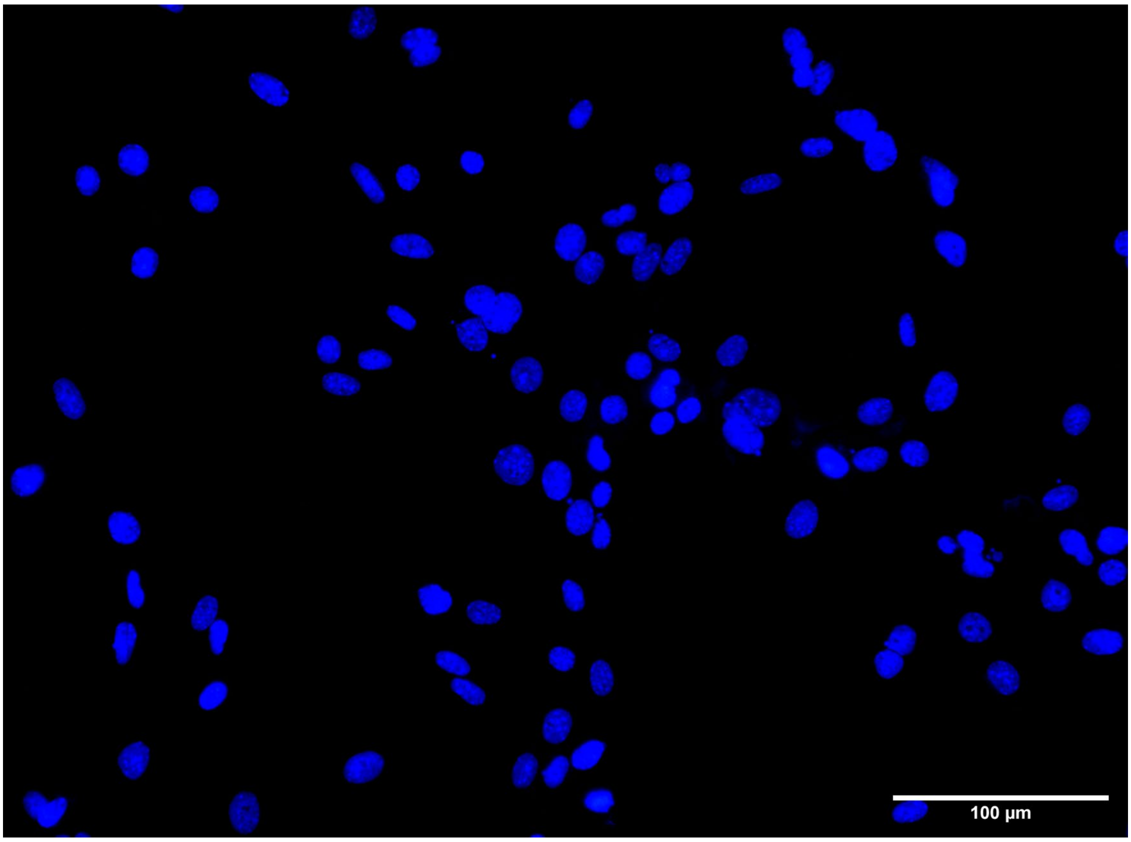

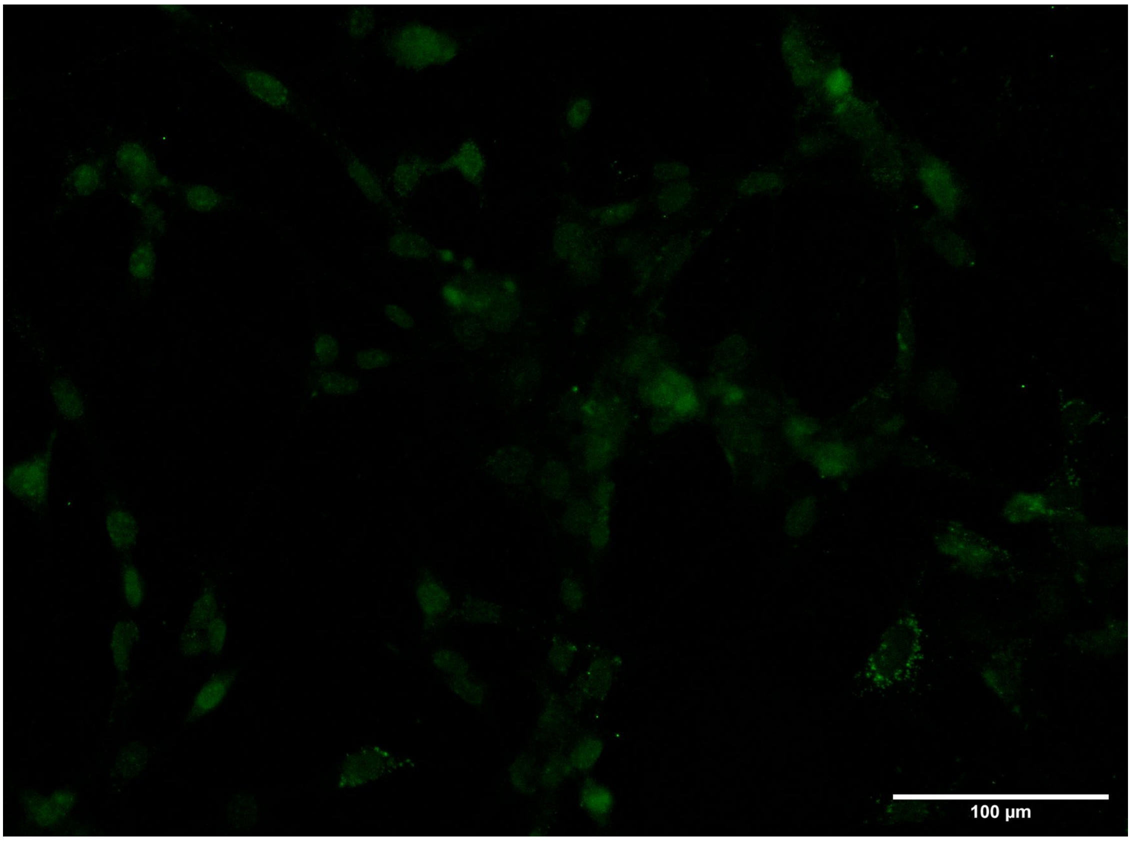

Figure S29. Immunofluorescence staining for p-STAT3 of irradiated OCYs co-cultured with CM-2 Gy + anti-CCL5 p-STAT3 (shown in green) and DAPI (shown in blue); Magnification = x200.
